# Long‐term effects of cholinesterase inhibitors and memantine on cognitive decline, cardiovascular events, and mortality in dementia with Lewy bodies: An up to 10‐year follow‐up study

**DOI:** 10.1002/alz.14118

**Published:** 2024-08-23

**Authors:** Hong Xu, Annegret Habich, Daniel Ferreira, Londos Elisabet, Eric Westman, Maria Eriksdotter

**Affiliations:** ^1^ Division of Clinical Geriatrics, Center for Alzheimer Research, Department of Neurobiology, Care Sciences and Society Karolinska Institutet Stockholm Sweden; ^2^ University Hospital of Psychiatry and Psychotherapy University of Bern Bern Switzerland; ^3^ Facultad de Ciencias de la Salud Universidad Fernando Pessoa Canarias Las Palmas España; ^4^ Institution of Clinical Sciences Lund University Malmö Sweden; ^5^ Theme Inflammation and Aging Karolinska University Hospital Stockholm Sweden

**Keywords:** cholinesterase inhibitors, dementia with Lewy bodies, memantine, MMSE, mortality

## Abstract

**INTRODUCTION:**

We aimed to assess the impact of cholinesterase inhibitors (ChEIs) and memantine on cognition, major adverse cardiovascular events (MACE) and mortality in dementia with Lewy bodies (DLB).

**METHODS:**

A total of 1,095 incident DLB patients from the Swedish Registry on cognitive/dementia disorders were included. Using an inverse probability of treatment weighting, the effect of initiating ChEI or memantine within 90 days of DLB diagnosis and nonuse was evaluated on cognitive trajectories and risks of MACE and death.

**RESULTS:**

The use of ChEIs significantly slowed cognitive decline at follow‐ups (Mini‐Mental State Examination [MMSE] ‐0.39 points/y; 95% confidence interval [CI], ‐0.96 to 0.18) compared to memantine (‐2.49 points/y; ‐4.02 to ‐0.97) and nonuse (‐2.50 points/y; ‐4.28 to ‐0.73). Treatment groups did not differ in MACE events. ChEI use was associated with lower risk of death in the first year after DLB diagnosis (adjusted hazard ratio [HR] 0.66, 95% CI 0.46, 0.94).

**DISCUSSION:**

Our findings illuminate the potential benefits of ChEI treatment in DLB patients.

**Highlights:**

- Cholinesterase inhibitors slow cognitive decline over a 5‐year follow‐up period when compared to both memantine treatment and nonuse in patients with dementia with Lewy bodies.
- Cholinesterase Inhibitors reduce risk of mortality within the initial year, but this effect is not sustained after 1 year in patients with dementia with Lewy bodies.

## 1 BACKGROUND

Lewy body disease, which includes dementia with Lewy bodies (DLB) and Parkinson's disease with and without dementia, is the second most common pathological cause of neurodegenerative disorders, following Alzheimer's disease (AD).^1^ DLB has been shown to account for approximately 10‐15% of dementia cases,^2^ with an incidence rate ranging from 1 to 5 per 1000 person/year.^3^ The global prevalence of DLB is projected to increase from 5.5 million individuals in 2020, 14 million by 2050.^4^ DLB is associated with faster cognitive and functional decline compared to AD.^5^ This leads to a notably worsening of quality of life in affected individuals when contrasted with AD patients.^6^ Furthermore, the direct economic burden associated with DLB is approximately twice that of AD when comparing the same number of subjects.^7^

To our knowledge, DLB lacks an approved treatment worldwide except in Japan, and clinical trials have proven unsuccessful in all disease‐modifying therapies (e.g., target a‐synuclein, incretin mimetic, and immuno‐therapy).^8^, ^9^ Most symptom‐modifying treatments are prescribed off‐label.^8^, ^9^ For instance, cholinesterase inhibitors (ChEIs, including donepezil, galantamine, and rivastigmine) and memantine are used to address cognitive symptoms. Clinical trials involving ChEIs in DLB have demonstrated improvements in cognition compared to placebo.^10^ A meta‐analysis encompassing ten trials indicated that the use of rivastigmine and donepezil resulted in an annual improvement of 1‐2.5 points on the Mini‐Mental State Examination (MMSE), while memantine showed no beneficial effect.^11^ Similarly, another meta‐analysis, which included fifteen trials supported the notion that ChEIs, but not memantine, led to cognitive improvement in patients with DLB.^12^ However, a recent network meta‐analysis involving eight randomized controlled trials showed no significant difference between ChEIs or memantine compared to placebo.^13^ It is noteworthy that most clinical trials for DLB have been limited to a maximum duration of 6 months to 1 year, leaving a paucity of knowledge regarding the long‐term effects of these treatments on cognition.

Apart from the beneficial effect of ChEIs on cognition, observational studies have indicated that ChEIs are associated with a decreased risk of myocardial infarction,^14^ heart failure,^15^ stroke,^16^ chronic kidney disease progression,^17^ and mortality.^14^, ^15^, ^16^, ^17^, ^18^, ^19^ In contrast, no effect of ChEIs was previously shown on adverse outcomes such as bradycardia or atrial‐ventricular block^19^ in patients with AD. The survival benefit of ChEIs has also been observed in a clinical trial in patients with Parkinson's disease with dementia.^20^ A recent population‐based study from the Cambridgeshire and Peterborough NHS Foundation Trust (CPFT) cohort reported that the use of ChEIs was associated with a reduced risk of mortality in individuals with DLB.^21^ Another cohort study from the National Alzheimer's Coordinating Center Uniform Data set (NACCUDS) showed that the use of ChEI is associated with a lower all‐cause mortality risk in a propensity score‐matched dementia cohort, including individuals with Lewy body disease.^22^ The potential explanation was that ChEIs act by inhibiting cholinesterase, leading to elevated acetylcholine levels in the synapses of both central and peripheral nervous system,^23^ with positive effects on cognitive function, amelioration of psychiatric symptoms, and activation of the cholinergic anti‐inflammatory pathway (CAP).^24^, ^25^, ^26^ Validation of these findings in other populations with differing characteristics is important to establish robust treatment recommendations in patients with DLB.

The goal of this study was to investigate the long‐term use of ChEIs or memantine for up to 10 years of follow‐up. The first aim was to evaluate the association of long‐term use of ChEIs or memantine with longitudinal MMSE trajectories. The second aim was to investigate the potential association of the use of ChEIs or memantine with the risk of major adverse cardiovascular events (MACE) and all‐cause mortality. Furthermore, we assessed different types and dosages of ChEIs to gain deeper insights into their potential effects on cognition.

## 2 METHODS

### 2.1 Data sources

We use data from the Swedish Registry on cognitive/dementia disorders (SveDem; www.svedem.se). SveDem is a web‐based registry started in 2007 with the aim to characterize and follow all individuals with dementia from the time of the dementia diagnosis in Sweden. The variables selected for the current study include patient demographics, MMSE scores, type of dementia, and treatment at diagnosis date and annual follow‐ups.^27^ For this study, we integrated SveDem with additional data from the National Patient Registry, which includes diagnoses from specialist clinics and hospitals, the Prescribed Drug Registry (where all prescribed drugs are recorded since 2006), and the Total Population and Causes of Death Registry.

In Sweden, individuals with dementia undergo a comprehensive diagnostic assessment according to the national guidelines of dementia care published by the Swedish Board of Health and Welfare^27^ to ensure they receive appropriate treatment and care tailored to their specific dementia diagnosis. Diagnoses are classified according to International Classification of Diseases, Tenth Revision (ICD‐10). Additionally, McKeith criteria^28^ was used for DLB, including the assessment of core clinical features such as progressive cognitive decline, cognitive fluctuations, visual hallucinations, rapid eye movement, and parkinsonism. Additional supportive features, including sleep behavior disorder and abnormal dopamine transporter imaging, may further support the diagnosis. Achieving an accurate diagnosis often requires a multidisciplinary approach involving neurologists, geriatricians, neuropsychologists, and other specialists.

There is no information on ethnicity in Sweden. Participants and their caregivers were informed verbally and in writing about SveDem and had the option to decline participation. However, the requirement of written consent for this study was waived due to the register data being pseudonymized before delivery to our research group. The regional ethics committee in Stockholm approved the study, which complied with the Declaration of Helsinki.^29^

### 2.2 Study population

**FIGURE 1 alz14118-fig-0001:**
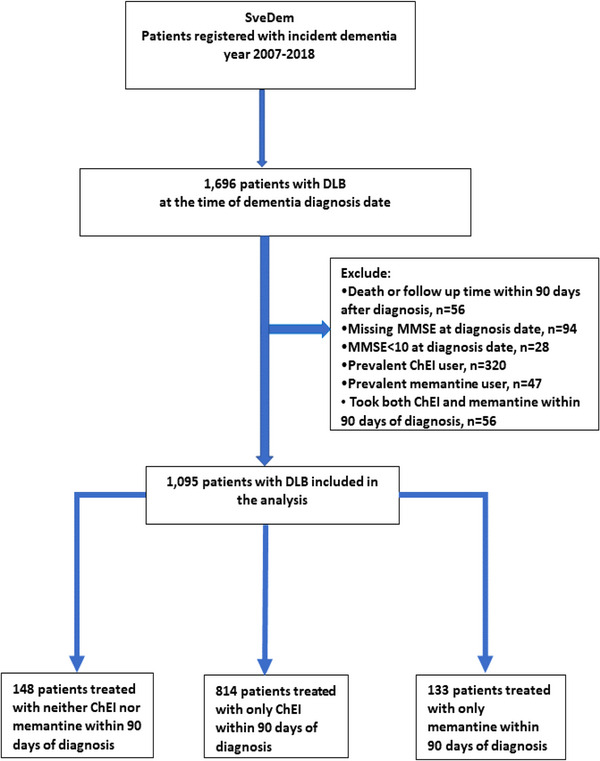
Flowchart. ChEI, acetylcholinesterase inhibitors; DLB, dementia with Lewy bodies; MMSE, Mini‐Mental State Examination.

We conducted a cohort study using a landmark design to compare cognition trajectories and adverse outcomes among patients diagnosed with DLB who were prescribed different treatments within 3 months of diagnosis. The treatments considered were ChEI alone or memantine alone, versus no treatment. To form our study population, we identified all patients who received an incident diagnosis of DLB and were registered in SveDem between May 1, 2007, and December 31, 2018 (*n* = 1,696). We excluded patients who were already on ChEI or memantine at the time of their dementia diagnosis (prevalent ChEI users, *n* = 320 or memantine users, *n* = 47), as well as those who started concomitantly with both therapies (*n* = 56). An additional 122 individuals were excluded because of missing MMSE scores at the time of diagnosis (*n* = 94) or with a baseline MMSE score < 10 (*n* = 28). The reason for this exclusion was that ChEIs in Sweden should be initiated in mild to moderate dementia (MMSE score ≥10), as well as to minimize baseline differences in MMSE among different treatment groups. The index date of the study and start of follow‐up was set 3 months after the incident dementia diagnosis, a clinically reasonable time at which ChEI or memantine therapy was initiated or not. Patients who died (*n* = 30) or ended the follow‐up within the first 3 months after the dementia diagnosis (*n* = 26) were also excluded from the analysis. After applying our inclusion and exclusion criteria, a total of 1,095 incident DLB patients constituted our study population (Figure 1).

**RESEARCH IN CONTEXT**

1. **Systematic review**: Currently, dementia with Lewy bodies (DLB) lacks approved treatments, leading to off‐label use of cholinesterase inhibitors (ChEIs) and memantine for symptom relief. However, the effectiveness of ChEIs in DLB remains uncertain due to inconsistent trial results and limited long‐term data. The goal of the study is to investigate the long‐term effects of ChEIs and memantine over 10 years, assessing their impact on cognitive decline, major cardiovascular events, and all‐cause mortality in DLB patients.
2. **Interpretation**: In this treatment‐weighted cohort study, the use of ChEIs in patients with DLB, compared to memantine treatment or nonuse, is associated with a reduced risk of cognitive decline over 5 years. No significant differences in major cardiovascular events were observed between all groups. However, ChEI use was associated with lower mortality at the first year of follow‐up, but not in longer follow‐up.
3. **Future directions**: Our findings shed light on the potential cognitive benefits of ChEI treatment in DLB patients. Further research is necessary to elucidate the underlying mechanisms and explore potential pleiotropic effects of ChEIs on long‐term outcomes.

### 2.3 Exposure

Study exposure was initiation of ChEIs therapy alone (donepezil, rivastigmine, or galantamine), or memantine alone versus no treatment within 3 months of the dementia diagnosis. Additionally, we collected data on the specific doses of ChEIs dispensation given to patients within the initial 3‐month period (in the subcohort of patients with ChEIs).

Our primary analysis used an intention‐to‐treat design, which means that we analyzed the outcomes based on the initial treatment group assignment, regardless of whether patients continued or discontinued the medication later. We assumed the study exposures to be constant until the end of follow‐up.[Fig alz14118-fig-0001]

### 2.4 Covariates

Covariates were defined at the index date and included calendar year of diagnosis, age, sex, MMSE score at diagnosis, whether the diagnosis was issued at a memory clinic or primary care, whether the patient was living alone or in a nursing home, dementia basic workups (clock test, blood test, MMSE test, and CT/MRI), comorbidities (alcohol abuse, history of acute kidney injury, atrial fibrillation, atrial‐ventricular block, bradycardia, malignant cancer within 5 years, cerebrovascular disease, congestive heart failure, chronic kidney disease, chronic pulmonary disease, depression, diabetes, fracture, hearing loss, hypertension, liver disease, myocardial infarction, peptic ulcer disease, peripheral vascular disease, rheumatic disease, smoking, and stroke), presence of a cardiac pacemaker and ongoing medications (angiotensin‐converting enzyme inhibitors/angiotensin receptor blockers [ACEI/ARBs], antidepressants, antipsychotics, antithrombotic, anxiolytics, β‐blockers, calcium channel blockers, diuretics, hypnotics, nonsteroidal anti‐inflammatory drugs, and statins). Definitions of comorbidities and medications are summarized in Supplement Table e‐1.

### 2.5 Outcomes and follow‐up periods

The first aim was to examine the trajectories of MMSE scores over 1‐5 years. The secondary aim was an occurrence of MACE events (defined as the composite of hospitalization of myocardial infarction or congestive heart failure or stroke) and all causes of death (Supplement Table e‐2) up to 10 years. Patients were annually followed from their index date (3 months after the DLB diagnosis, T_0_) until migration from the region, death, or the end of follow‐up (December 31, 2018), whichever happened first. This gave us the unique opportunity to assess our outcomes over a period of up to 10 years. To better understand the temporality of our findings, we stratified analyses into shorter‐term follow‐up periods (1, 2, and 3 years) and longer‐term follow‐up periods (3‐10 years) based on the median follow‐up time.

### 2.6 Statistical analyses

Continuous variables are reported as either mean with standard deviation (SD) or median with interquartile range (IQR). Categorical variables are presented as percentages. As per the study design, all the study covariates were complete; however, this does not preclude missing due to no clinical record. To account for confounding by indication, we employed the inverse probability of treatment weighting (IPTW) method.^30^ This method involved estimating the probability of initiating different treatments based on all the study covariates mentioned earlier. Each patient in every treatment group was assigned a weight of 1 divided by their propensity score (PS). Weights were stabilized by adding the marginal probability of the received treatment to the numerator of the weights. Weighting was considered appropriate if the standardized mean difference (SMD) among treatment groups was ≤0.1.

To address our first aim, which involved MMSE score trajectories, we used the inverse probability of treatment weighted cohort. We applied mixed‐effects models to incorporate data from all visits throughout the follow‐up period, spanning from year 1 to 5, with a random intercept. The mixed‐effects model included treatments, follow‐up year (in a cubic spline term), total number of performed MMSE assessments, and the interaction between treatment and follow‐up year to estimate the differences in the annual MMSE change attributed to different treatments. Furthermore, we considered the potential impact of attrition due to dropout or the presence of a competing risk (e.g., death) during the follow‐up. To address this concern, we applied inverse probability of censoring weighting in conjunction with the mixed‐effect model.^31^ This approach allowed us to appropriately handle potential biases arising from attrition in our analysis. Finally, we calculated means and 95% confidence intervals (CIs) using the estimated marginal means of the MMSE trajectories and conducted adjusted multiple pairwise comparisons within groups to assess the significance of differences in treatment groups at each year visit.

To address our second aim of MACE and mortality, we calculated the number of events and incidence rates per 1000 person‐years with corresponding 95% CIs. To estimate hazard ratios (HRs) and 95% CIs between treatments and outcomes, we first performed a weighted Cox regression model. The proportional hazards assumption was checked with the Schoenfeld residuals test. In the case of violation of the proportionality assumption for certain treatments, we applied weighted flexible parametric survival models (Royston‐Parmar models) to estimate HRs and their associated 95% CI at 1, 2, 3, and 10 years of follow‐up. We also evaluated the consistency of effect across ChEI types on outcomes (donepezil, rivastigmine, or galantamine).

### 2.7 Subgroup and sensitivity analysis

Subgroup analyses were performed to test for potential age (using median split; ≤77 vs. > 77 years) or sex (men vs. women) effects on the association between treatment and MMSE trajectories.

In addition, we conducted various sensitivity analyses to test the robustness of our results:

To mitigate potential deviations from the initial treatment, including crossover situations where some patients were taking ChEI or memantine after a long‐term DLB diagnosis, we addressed this by censoring the data at the point of treatment discontinuation or transition to one of the comparator drugs. This approach allowed us to estimate the as‐treated effect. ii) We evaluated the association between the maintained dose of ChEIs in the patients with ChEI treatment and study outcomes in the subcohort of only ChEI users (*n* = 814). We used the Defined Daily Dosages (DDD) for each ChEI. The DDDs represent the assumed average maintenance dose per day for a drug used for its main indications in adults. The value of DDD is established by the World Health Organization International Working Group for Drug Statistics Methodology and is a widely accepted method for measuring drug utilization and exposure.^32^ We modeled ChEI doses as a continuous exposure for increasing doses in a cubic spline with outcomes. 1 DDD is equal to donepezil 7.5 mg, rivastigmine 9 mg, or galantamine 16 mg.

All analyses were performed using R 3.4.3 software (The R Project for Statistical Computing, Vienna, Austria) and Stata version 17.0 (StataCorp, College Station, TX).

## 3 RESULTS

### 3.1 Patient characteristics

We included a total of 1,095 individuals with incident DLB between May 1, 2007, and December 31, 2018. The mean age was 76.7 ± 7.0 years, 38% were women, and the mean MMSE score was 21.9 ± 4.4 points at baseline. A total of 814 individuals started on ChEIs alone within 3 months (69% received rivastigmine, 21% donepezil, and 10% galantamine), while 133 individuals started on memantine alone, and 148 individuals did not receive any of these medications (patient selection flow chart in Figure 1). Baseline characteristics of the study cohort are detailed in Table 1. When compared to nonusers and memantine users, individuals who started on ChEIs tended to be younger and were less likely to be in nursing home care. ChEI users showed a lower prevalence of comorbidities and less use of concomitant medication compared to nonusers and memantine users. All characteristics were well balanced after weighting with SMD ≤0.1 (Supplement Figure e‐1). In addition, no significant differences in baseline characteristics were observed between different ChEIs (donepezil, rivastigmine, and galantamine) (Supplement Table e‐3). After weighting, the characteristics of individual ChEI users were balanced when compared to nonusers or memantine users (Supplement Figure e‐2).

**TABLE 1:** Baseline characteristics stratified by treatment status within 3 months from an incident diagnosis of dementia with Lewy bodies.

Parameter	None (*n* = 148)	Memantine alone (*n* = 133)	ChEI alone (*n* = 814)	*p*‐value
**Demographics**				
Age, mean (SD)	77.4 (8.2)	78.5 (6.7)	76.2 (6.7)	<0.001
Female	66 (44.6%)	52 (39.1%)	300 (36.9%)	0.20
MMSE baseline, mean (SD)	21.3 (5.0)	21.5 (4.6)	22.1 (4.3)	0.07
MMSE strata				0.17
10‐19	47 (31.8%)	45 (33.8%)	205 (25.2%)	–
20‐24	60 (40.5%)	49 (36.8%)	357 (43.9%)	–
> 25	41 (27.7%)	39 (29.3%)	252 (31.0%)	–
Specialist visit	119 (80.4%)	126 (94.7%)	762 (93.6%)	<0.001
Living alone	65 (43.9%)	35 (26.3%)	279 (34.3%)	0.01
Nursing home	22 (14.9%)	26 (19.5%)	54 (6.6%)	<0.001
**Comorbidities**				
Chronic kidney disease	5 (3.4%)	6 (4.5%)	27 (3.3%)	0.78
Hypertension	66 (44.6%)	61 (45.9%)	323 (39.7%)	0.26
Diabetes	25 (16.9%)	12 (9.0%)	100 (12.3%)	0.13
Myocardial Infarction	11 (7.4%)	11 (8.3%)	63 (7.7%)	0.97
Congestive heart failure	18 (12.2%)	15 (11.3%)	59 (7.2%)	0.06
Atrial fibrillation	30 (20.3%)	35 (26.3%)	119 (14.6%)	0.01
Cardiac pacemaker	5 (3.4%)	3 (2.3%)	34 (4.2%)	0.54
Atrial‐Ventricular block	1 (0.7%)	5 (3.8%)	19 (2.3%)	0.22
Bradycardia	3 (2.0%)	3 (2.3%)	9 (1.1%)	0.43
Peripheral vascular disease	6 (4.1%)	6 (4.5%)	33 (4.1%)	0.97
Cerebrovascular diseases	29 (19.6%)	29 (21.8%)	125 (15.4%)	0.11
Stroke	17 (11.5%)	13 (9.8%)	61 (7.5%)	0.22
Chronical pulmonary disease	15 (10.1%)	11 (8.3%)	65 (8.0%)	0.68
Rheumatic diseases	6 (4.1%)	1 (0.8%)	34 (4.2%)	0.15
Peptic ulcers disease	6 (4.1%)	8 (6.0%)	20 (2.5%)	0.07
Malignant Cancer within 5 years	13 (8.8%)	18 (13.5%)	78 (9.6%)	0.32
Liverdisease	2 (1.4%)	2 (1.5%)	7 (0.9%)	0.71
Alcohol abuse	4 (2.7%)	1 (0.8%)	14 (1.7%)	0.46
Fractures	41 (27.7%)	41 (30.8%)	194 (23.8%)	0.17
Hearing loss	15 (10.1%)	24 (18.0%)	83 (10.2%)	0.03
Depression	22 (14.9%)	12 (9.0%)	86 (10.6%)	0.23
**Medication**				
ACEI/ARB	55 (37.2%)	45 (33.8%)	269 (33.0%)	0.62
β‐blocker	57 (38.5%)	55 (41.4%)	246 (30.2%)	0.01
Calcium channel blocker	48 (32.4%)	36 (27.1%)	181 (22.2%)	0.02
Diuretics	48 (32.4%)	44 (33.1%)	230 (28.3%)	0.36
Statins	50 (33.8%)	45 (33.8%)	281 (34.5%)	0.98
NSAIDs	18 (12.2%)	15 (11.3%)	93 (11.4%)	0.96
Antithrombotic	100 (67.6%)	91 (68.4%)	539 (66.2%)	0.86
Antixiolytics	31 (20.9%)	43 (32.3%)	175 (21.5%)	0.02
Hypnotics	38 (25.7%)	42 (31.6%)	204 (25.1%)	0.28
Antipsychotics	31 (20.9%)	29 (21.8%)	151 (18.6%)	0.58
Antidepressants	64 (43.2%)	56 (42.1%)	328 (40.3%)	0.76
**ChEI type**				–
Donepezil			174 (21.4%)	
Rivastigmine			555 (68.2%)	
Galantamine			85 (10.4%)	
**Dementia diagnosis year**				0.11
2007	0 (0.0%)	0 (0.0%)	13 (1.6%)	
2008	6 (4.1%)	2 (1.5%)	37 (4.5%)	
2009	11 (7.4%)	4 (3.0%)	76 (9.3%)	
2010	10 (6.8%)	9 (6.8%)	75 (9.2%)	
2011	9 (6.1%)	11 (8.3%)	68 (8.4%)	
2012	12 (8.1%)	15 (11.3%)	82 (10.1%)	
2013	11 (7.4%)	15 (11.3%)	79 (9.7%)	
2014	17 (11.5%)	19 (14.3%)	95 (11.7%)	
2015	17 (11.5%)	14 (10.5%)	86 (10.6%)	
2016	19 (12.8%)	18 (13.5%)	86 (10.6%)	
2017	26 (17.6%)	14 (10.5%)	78 (9.6%)	
2018	10 (6.8%)	12 (9.0%)	39 (4.8%)	

Abbreviations: ACEi, angiotensin‐converting enzyme inhibitors; ARB, angiotensin receptor blockers; ChEI, acetylcholinesterase inhibitors; MMSE, Mini‐Mental State Examination; NSAIDs, nonsteroidal anti‐inflammatory drugs; SD, standard deviation.

### 3.2 ChEIs, memantine, and MMSE trajectories

**FIGURE 2 alz14118-fig-0002:**
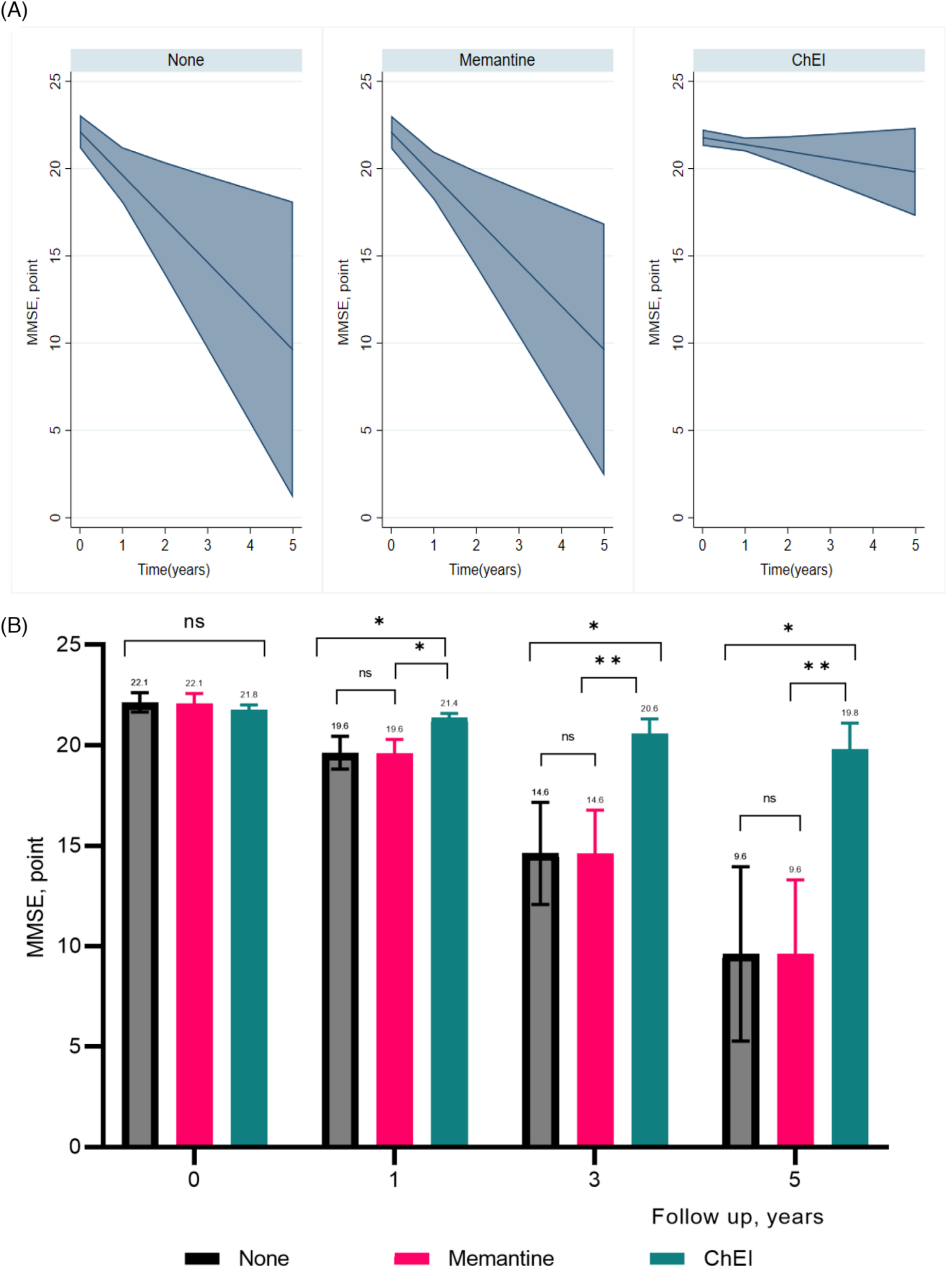
Mixed model output of estimated cognition trajectories by treatment status: (A) slope of MMSE and (B) MMSE score comparisons between treatments at different timepoints. Significantly increased MMSE scores in the ChEI users compared with nonusers or memantine users are shown. The MMSE changes over time in memantine is not significantly different from nonusers at any time point. MMSE estimation is obtained in inverse probability of treatment‐weighted cohort, additionally adjusted with inverse probability of censoring weighting. The mixed model included treatments, visit time (year by using splines), treatment by visit time, number of MMSE measurements with an unstructured covariance matrix within treatment group for a repeated‐measures covariance structure (random intercepts). ChEI, acetylcholinesterase inhibitors; MMSE, Mini‐Mental State Examination. *p < 0.05, **p < 0.01, ***p < 0.001.

Overall, there was no difference in MMSE scores among the three groups at baseline after weighting. During follow‐up, the difference in MMSE at each year visit differed significantly between ChEI users and nonusers, as well as between ChEI users and memantine users, but did not differ between memantine users and nonusers. ChEI users alone showed significantly slower cognitive decline (MMSE slope, −0.4 points/year, 95% CI: −0.96, 0.18) compared to nonusers (MMSE slope, −2.5 points, 95% CI: −4.28, −0.73) and memantine users (MMSE slope, −2.49 points, 95% CI: −4.02, −0.97) (Table 2 and Figure 2). Of the ChEIs, the difference in MMSE at each yearly visit significantly differed between donepezil, galantamine and nonusers, but not rivastigmine users and nonusers. There were no significant differences between donepezil, rivastigmine, and galantamine, except between galantamine and donepezil at year 5 visit. Galantamine (MMSE slope, 0.32 points/year, 95% CI: −0.97, 1.62) and donepezil (MMSE slope, 0.07 points/year, 95% CI: −1.01, 1.16) but not rivastigmine were significantly associated with slower cognitive decline compared to nonusers at each year visit (Supplement Table e‐4 and Supplement Figure e‐3A and e‐3B).

**TABLE 2:** Mixed model output of estimated MMSE trajectories by treatment status.

	MMSE estimation, β coefficient (95% CI)			Difference, β coefficient (95% CI)		
Treatment status	None (*n* = 148)	Memantine alone (*n* = 133)	ChEI alone (*n* = 814)	Memantine vs none	ChEI vs none	ChEI vs memantine
Baseline	22.13 (21.17 to 23.08)	22.09 (21.14 to 23.04)	21.78 (21.31 to 22.24)	−0.04 (−1.36,1.28)	−0.35 (−1.38,0.67)	−0.31 (−1.35,0.72)
Year 1	19.63 (18.03 to 21.23)	19.60 (18.22 to 20.97)	21.38 (20.98 to 21.79)	−0.03 (−2.14,2.07)	1.76 (0.10,3.41)*	1.79 (0.35,3.22)*
Year 2	17.12 (13.88 to 20.37)	17.10 (14.36 to 19.85)	20.99 (20.12 to 21.80)	−0.02 (−4.27,4.23)	3.87 (0.50,7.23)*	3.89 (1.00,6.77)**
Year 3	14.62 (9.64 to 19.60)	14.61 (10.38 to 18.83)	20.60 (19.18 to 22.01)	−0.01 (−6.54,6.51)	5.97 (0.80,11.15)*	5.99 (1.53,10.44)**
Year 4	12.12 (5.39 to 18.85)	12.11 (6.39 to 17.84)	20.20 (18.23 to 22.17)	−0.01 (−8.84,8.83)	8.08 (1.07,15.10)*	8.09 (2.04,14.14)**
Year 5	9.61 (1.12 to 18.11)	9.62 (2.38 to 16.86)	19.81 (17.28 to 22.34)	0.00 (−11.16,11.16)	10.19 (1.33,19.06)*	10.19 (2.53,17.85)**
MMSE slope	−2.50 (−4.28 to −0.73)	−2.49 (−4.02 to −0.97)	−0.39 (−0.96 to 0.18)	0.01 (−2.33 to 2.35)	2.11 (0.25 to 3.97)*	2.10 (0.48 to 3.72)*

*Note*: Estimation is obtained in inverse probability of treatment weighted cohort, additionally adjusted with inverse probability of censoring weighting (IPCW, we considered the potential effects of general attrition from those losses to follow‐up due to dropout or to the presence of a competing risk of death. The cohort was weighted for the following covariates: calendar year of diagnosis, age, sex, MMSE score at diagnosis, whether the diagnosis was issued at a memory clinic, whether the patient was living alone or in a nursing home, dementia basic workups (clock test, blood test, MMSE test and CT/MRI), comorbidities (alcohol abuse, acute kidney injury, atrial fibrillation, AV block, bradycardia, cancer, cerebrovascular disease, congestive heart failure, chronic kidney disease, chronic pulmonary disease, depression, diabetes, fracture, hearing loss, hypertension, liver disease, myocardial infarction, peptic ulcer disease, peripheral vascular disease, rheumatic disease, smoking, and stroke), presence of a cardiac pacemaker and ongoing medications (angiotensin‐converting enzyme inhibitors/angiotensin receptor blockers (ACEI/ARBs), antidepressants, antipsychotics, antithrombotic, anxiolytics, β‐blockers, calcium channel blocker, diuretics, hypnotics, nonsteroidal anti‐inflammatory drugs, and statins).

The mixed model included treatments, follow‐up time (year by using splines), treatment by follow‐up year, number of performed MMSE assessments with an unstructured covariance matrix within treatment group for a repeated‐measures covariance structure (random intercepts).

Abbreviations: ChEI, acetylcholinesterase inhibitors; CI, confidence interval; MMSE, Mini‐Mental State Examination.

*p < 0.05, **p < 0.01, ***p < 0.001.

The initial ChEI dose and MMSE trajectories are graphically represented in Supplement Figure e‐4. In ChEI users, a significant increase in MMSE over time was found with > 1DDD (i.e., 7.5 mg donepezil, 16 mg galantamine or 9 mg of rivastigmine). Characteristics according to DDD of ChEIs are shown in Supplemental Table e‐5. Individuals who were older, living alone, and had prescriptions of hypnotics, antipsychotics, and antidepressants were less likely to be treated with higher dosages of ChEIs. Interestingly, 62% of rivastigmine users did not reach the optimal dose (1 DDD), especially when compared to users of donepezil and users of galantamine, where 39% and 48% did not reach therapeutic dose.[Fig alz14118-fig-0002]

### 3.3 ChEIs, memantine, all‐cause mortality, and MACE events

Over an average follow‐up period of 3 (interquartile range 1.5‐4.1, range 0.2‐10) years, there were a total of 106 (10%) hospitalizations for MACE and 610 (56%) deaths observed in the study population. The cumulative incidence and hazard ratio curves are shown in Figure 3. ChEI users exhibited the lowest mortality rate compared to nonusers and memantine users in the initial year following DLB diagnosis (log‐rank *p* = 0.001). In comparison to nonuse, ChEI use was associated with a lower risk of death at the 1‐year follow‐up (adjusted HR [aHR] 0.66; 95% CI: 0.46‐0.94). However, this beneficial outcome diminished over time and a trend toward increasing risk was observed in longer‐term follow‐up. Memantine did not show any beneficial effect on mortality.

FIGURE 3Cumulative incidence (A) and hazard ratio with 95% CIs (B) for all‐cause mortality and MACE (composite of myocardial infarction, congestive heart failure, or stroke) for ChEIs and memantine versus nonuse. ChEI, acetylcholinesterase inhibitors; CIs, confidence intervals; MACE, major adverse cardiovascular events.

**Figure:**
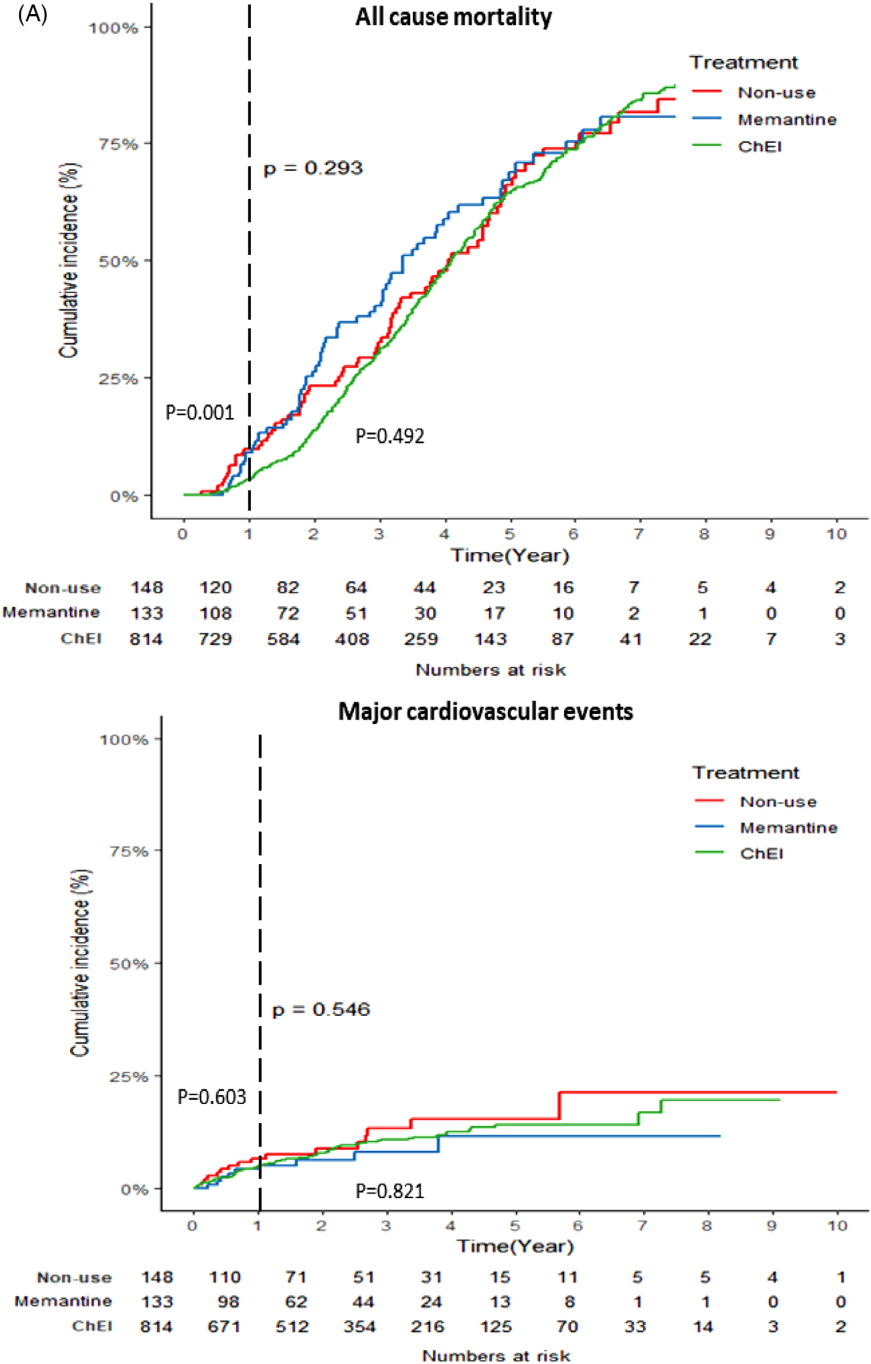

**Figure:**
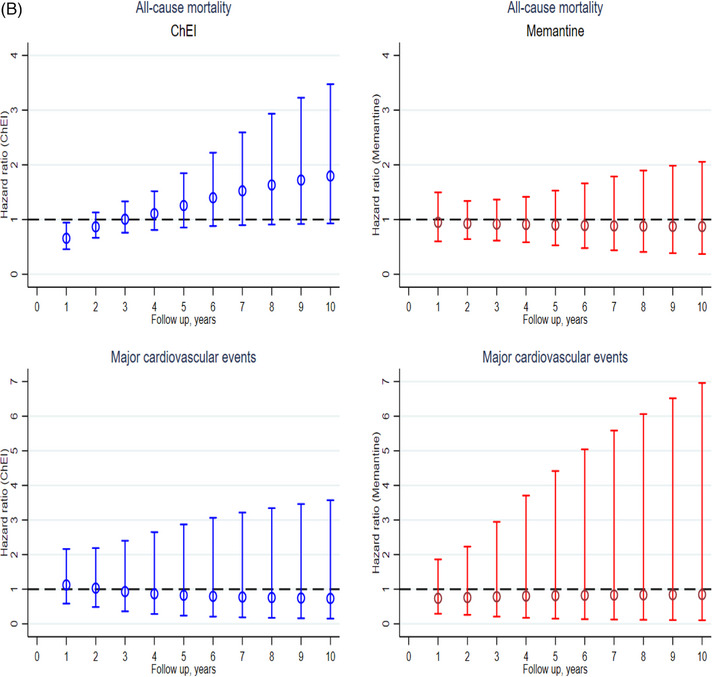

Users of donepezil, rivastigmine, and galantamine showed a lower mortality rate compared to nonusers and memantine users (log‐rank *p* = 0.001) at the 1‐year of follow‐up. A significant reduction in the hazard ratio for mortality was observed with rivastigmine at 1‐year (0.65, 0.43‐0.97). Additionally, we found that the risk of death with donepezil (0.61, 0.36‐1.07) and galantamine (0.50, 0.33‐1.04) was also reduced at 1—year, but it did not reach statistical significance. No effect on mortality with memantine use was found (Supplement Table e‐6 and Supplement Figure e‐5).

None of the treatments showed a significant association with hospitalization for MACE in both shorter and longer time periods, as shown in Table 3 and Figure 3. We observed similar results among different ChEIs with regard to MACE (Supplement Table e‐6 and Supplement Figure e‐5).

**TABLE 3:** Number of events, incidence rates, and adjusted HRs for the association between treatment status and adverse clinical outcomes

Parameter	Events	Person time, years	Overall incidence rate per 1000 py (95% CI)**^a^**	HR**^b^** (95% CI) at year 1	HR**^b^** (95% CI) at year 2	HR**^b^** (95% CI) at year 3	HR**^b^** (95% CI) at year 10
**Hospitalization due to MACE**							
None (*n* = 148)	16 (11%)	371.98	43.01 (26.35 to 70.21)	Ref	Ref	Ref	Ref
Memantine (*n* = 133)	9 (7%)	317.37	28.36 (14.75 to 54.50)	0.74 (0.29,1.86)	0.76 (0.26,2.23)	0.78 (0.21,2.95)	0.84 (0.10,6.96)
ChEI (*n* = 814)	81 (10%)	2390.10	33.89 (27.26 to 42.14)	1.12 (0.59,2.16)	1.03 (0.48,2.19)	0.93 (0.36,2.40)	0.73 (0.15,3.57)
**Deaths**							
None (*n* = 148)	77 (52%)	406.64	189.36 (151.45 to 236.75)	Ref	Ref	Ref	Ref
Memantine (*n* = 133)	69 (52%)	329.13	209.65 (165.58 to 265.44)	0.95 (0.60,1.50)	0.93 (0.64,1.33)	0.92 (0.62,1.36)	0.87 (0.37,2.05)
ChEI (*n* = 814)	464 (57%)	2504.95	185.23 (169.12 to 202.88)	0.66* (0.46,0.94)	0.86 (0.67,1.13)	1.00 (0.76,1.33)	1.79 (0.93,3.47)

Abbreviations: ChEI, acetylcholinesterase inhibitors; CI, confidence interal; HR, hazard ratio.

^a^ Incidence rates are presented as number of events per 1000 patient‐years in unweighted cohort.

^b^ Hazard ratio is obtained in the inverse probability of treatment‐weighted cohort with flexible parametric survival model. The cohort was weighted for the following covariates: calendar year of diagnosis, age, sex, MMSE score at diagnosis, whether the diagnosis was issued at a memory clinic, whether the patient was living alone or in a nursing home, dementia basic workups (clock test, blood test, MMSE test and CT/MRI), comorbidities (alcohol abuse, acute kidney injury, atrial fibrillation, AV block, bradycardia, cancer, cerebrovascular disease, congestive heart failure, chronic kidney disease, chronic pulmonary disease, depression, diabetes, fracture, hearing loss, hypertension, liver disease, myocardial infarction, peptic ulcer disease, peripheral vascular disease, rheumatic disease, smoking, and stroke), presence of a cardiac pacemaker and ongoing medications (angiotensin‐converting enzyme inhibitors /angiotensin receptor blockers (ACEI/ARBs), antidepressants, antipsychotics, antithrombotic, anxiolytics, β‐blockers, calcium channel blocker, diuretics, hypnotics, nonsteroidal anti‐inflammatory drugs, and statins).

### 3.4 Sensitivity and subgroup analysis

After censoring 57 nonusers who initiated ChEI or memantine during follow‐up, and 414 ChEI and memantine who switched treatment or discontinued during follow‐up, we observed similar results for MMSE trajectories, MACE, and all‐cause mortality compared to our main analysis (Supplementary Table e‐7 and e‐8).

Subgroup analyses revealed the consistent cognitive benefits associated with ChEI usage, irrespective of age or gender, as indicated by the MMSE trajectories. Specifically, individuals using ChEI showed a significant slowdown in cognitive decline compared to both nonusers and those taking memantine. This effect was observed in both younger and older age groups (≤77 vs. > 77 years) as well as across both genders (Supplement Figure e‐6).

## 4 DISCUSSION

In this large, longitudinal, nationwide DLB cohort, ChEI use slowed cognitive decline compared to memantine and nonuse. A dose‐response relationship suggested higher ChEI doses provided greater cognitive benefits. Significantly improved MMSE scores were noted for donepezil and galantamine, but not rivastigmine. Memantine showed no significant cognitive effect. Contrary to our prior findings in AD and our initial hypothesis, ChEI in DLB did not reduce major adverse cardiovascular events or mortality risk in the long term. ChEI treatment showed a significantly lower mortality risk, which was only evident during the first year of follow‐up. Among the ChEIs, only rivastigmine showed a significant beneficial effect. We found that the risk was also reduced between donepezil and galantamine, but it did not reach statistical significance. These findings shed light on the potential cognitive benefits of ChEI treatment in patients with DLB but also highlight challenges.

Our study found significantly slower cognitive decline in patients using ChEI, consistent with several clinical trials and meta‐analyses.^12^, ^33^, ^34^ For instance, a randomized, placebo‐controlled trial in Japan with DLB patients (*n* = 140) showed donepezil (5 mg or 10 mg) improved MMSE scores by 2.4‐3.8 points after 12 weeks, with positive changes in behavioral symptoms and caregiver burden.^35^ The cognitive benefits of donepezil persisted in an open‐label extension,^36^ whereas a phase III study only confirmed benefits for the 10 mg dose.^37^ An open‐label, multicenter study with 50 DLB patients treated with galantamine (24 mg for 24 weeks) showed stable MMSE scores.^38^ Our findings align with these results, indicating donepezil and galantamine slow cognitive decline. A large randomized, double‐blind, placebo‐controlled study (*n* = 120) with DLB patients receiving rivastigmine (6‐12 mg for 20 weeks) found improvements in computerized cognitive tasks but no difference in MMSE scores, similar to our results.^39^ One possible explanation is that the majority of the rivastigmine users (in contrast to users of donepezil and galantamine) in our cohort did not achieve the recommended dose. Additionally, two double‐blind, placebo‐controlled trials with DLB and Parkinson's disease dementia patients (*n* = 72 and *n* = 199) found no cognitive benefits from memantine (20 mg for 24 weeks), which our study also confirmed.^40^, ^41^

Our findings, however, disagree with other trials/studies. For instance, a recent network meta‐analysis, which included eight randomized controlled trials, did not demonstrate any significant differences between the effects of various dosages of donepezil (3 mg, 5 mg, and 10 mg), rivastigmine (not mentioned dose in the meta‐analysis), or memantine on MMSE changes compared to placebo.^13^ Subgroup analysis of the NACCUDS cohort showed that ChEI use did not exhibit significant cognitive benefits compared with nonusers in Lewy body disease, which also includes Parkinson's disease dementia, and may have a different response to ChEI treatment.^22^ The observed disparity between these reports and our study might be explained by differences in study design (clinical trial vs. cohort study), as well as variations in ages, genders distributions, diagnostic groups, patient comorbidities, and treatment strategies between our cohort and the subcohort from NACCUDS. However, we believe that these previous studies included insufficient number of patients with shorter follow‐up periods, which could have limited their power to detect treatment effects on cognitive performance.

The potential mechanism underlying the improvement of MMSE scores with ChEI treatment in DLB is associated with the cholinergic system. Previous studies demonstrated that DLB patients exhibit a pronounced impairment of the cholinergic system. Specifically, atrophy in the basal forebrain, particularly in the nucleus basalis of Meynert and its associated cholinergic projections, has been shown already in the prodromal stages of DLB.^42^ Furthermore, impairment of cholinergic system has been associated with cognitive functioning in individuals with prodromal DLB.^43^ Similarly, DLB patients' performance on the MMSE correlates with the degree of impairment in their cholinergic circuits, as assessed through transcranial magnetic stimulation.^44^ Of note, associations between volume loss in the basal forebrain and cognitive impairments were repeatedly shown to be stronger in DLB compared to AD patients.^45^, ^46^ This observation, coupled with higher gene expression levels of acetylcholinesterase in DLB as compared with AD patients,^47^ led to the hypothesis that treatment with ChEI would be more effective in slowing cognitive decline in DLB compared to AD.^48^ In fact, DLB patients without AD co‐pathology were more likely to benefit from ChEIs^49^ than DLB patients with AD co‐pathology. These findings suggest that targeting the cholinergic system may hold greater promise in delaying cognitive decline in DLB, particularly in cases without AD co‐pathology.

Our study showed a reduced mortality risk with ChEI use at the first‐year follow‐up, but we cannot confirm the long‐term survival benefits reported by Chen et al.^21^ Their study of 592 DLB patients reported 424 deaths over 12 years and found a favorable association with ChEIs. Differences between their study and ours may be due to factors like prescription patterns (71% ChEI alone in Sweden vs. 17% in the United Kingdom) and combination treatments (5% vs. 46%). Additionally, our cohort was younger (77 vs. 82 years) and had fewer females (38% vs. 50%), which might explain the discrepancies.^21^ The NACCUDS cohort showed that ChEI use was associated with a decrease in mortality risk, with an HR of 0.63 in Lewy body disease (*n* = 124 after PS matching).^22^ A previous study showed that in patients with DLB and Parkinsons's disease with dementia, younger age at diagnosis, female sex, lower MMSE, and apolipoprotein E (APOE) ε4 carriership were linked to excessive mortality relative to the general population.^50^ The nonsignificant mortality risk reduction by the ChEIs in DLB on long‐term follow‐up shown here is in contrast to the long‐term mortality risk reduction in the AD population shown by our group^14^, ^16^, ^17^ and others.^51^ The absence of long‐term survival benefits might be attributable to the strong autonomic dysfunction, evident in persons with DLB and repeatedly observed shorter survival in patients with DLB compared to AD.^52^, ^53^, ^54^ In fact, a survey among caregivers and relatives of DLB patients revealed that a majority of patients died within 5 years.^55^ Patients with DLB and AD also differ regarding causes of death with nervous system‐related and respiratory causes being more prevalent in DLB.^54^

The strengths of our study include a large sample size and up to 10 years of follow‐up, enhancing validity through real‐world data from standard patient registration. Nevertheless, we must acknowledge certain limitations. First, given the observational nature of our study design, causality cannot be directly inferred, and we recognize the potential for residual and unknown confounding factors. However, to mitigate this concern, we employed inverse probability treatment weighted cohort analysis to control for unbalanced confounders. Second, throughout the entire follow‐up duration, patients were considered exposed based on their treatment status at the study's entry. This approach was adopted to emulate the intention‐to‐treat design employed in clinical trials, ensuring a conservative estimation of the effects of ChEIs and/or memantine on cognition. However, some patients may have stopped/switched treatment over time. To account for this, we estimated an as‐treated effect (per protocol design) with similar results as in our main analyses. Third, we lacked information on patients' lifestyle habits, frailty, severity of the comorbidities, blood pressure, AD co‐pathology information of DLB, all of which could influence our findings. These factors should be considered in future studies for a more comprehensive evaluation. Fourth, a limitation of our study is the inherent challenge of accurately diagnosing DLB. Although DLB diagnosis was based on national guidelines and the McKeith criteria, this procedure may not be entirely effective in identifying all cases with DLB. Fifth, since our data were derived from real‐world clinical practice, variations in the frequency of MMSE measurements were observed among individuals. Nonetheless, to address this concern, we applied inverse probability of censoring weighting to adjust the estimates, thus attenuating potential biases arising from dropout.^31^ Given the potential limitations of the MMSE in assessing executive function and attention, the interpretation of our MMSE results needs to be considered in that context.^56^ Last, the number of participants remaining in our cohort at the 10‐year follow‐up is quite low; therefore, interpretation of the results should be done with caution.

In conclusion, this study demonstrates that the use of ChEIs in patients with DLB may reduce the risk of cognitive decline compared to memantine treatment or no treatment. ChEI treatment in DLB is associated with a lower risk of death early after diagnosis. Furthermore, we found no beneficial effects of memantine in our study. Future studies are needed to better elucidate these mechanisms. Clinical trials should be conducted for longer than 2 years of follow‐up to assess long‐term outcomes such as cardiovascular events and mortality. Future studies should also consider 3‐5 years of long‐term follow‐up, starting from prodromal DLB (mild cognitive impairment with Lewy bodies MCI‐LB), to provide comprehensive coverage of long‐term observations. Our study sheds light on the use of ChEI in individuals with DLB and supports a change in guideline recommendations regarding ChEI in DLB.

## AUTHOR CONTRIBUTION

Hong Xu, Daniel Ferreira, and Maria Eriksdotter jointly developed the study concept and design. H.X. contributed to data analysis, while Hong Xu, Annegret Habich, Daniel Ferreira, Londos Elisabet, Eric Westman, and Maria Eriksdotter contributed to writing the report. HX and ME provided study materials. All authors contributed to data interpretation, critical revision of the report, and final approval. Hong Xu, Daniel Ferreira, and Maria Eriksdotter obtained funding. Hong Xu and Maria Eriksdotter take responsibility for all aspects of the report, and all authors take responsibility for their contributions.

## CONFLICT OF INTEREST STATEMENT

The authors declare no conflicts of interest. Author disclosures are available in the supporting information.

## CONSENT STATEMENT

Data were extracted anonymized from electronic records. The Swedish Ethical Review Authority waived patient consent for this study.

## Supporting information

Supporting Information

Supporting Information
