# Supplementary material for: Long‐term effects of cholinesterase inhibitors and memantine on cognitive decline, cardiovascular events, and mortality in dementia with Lewy bodies: An up to 10‐year follow‐up study
Source: Alzheimers Dement. 2024 Aug 23;20(10):6740–54. doi: 10.1002/alz.14118 (PMC11485406; doi:10.1002/alz.14118)

**Long-Term Effects of Cholinesterase Inhibitors and Memantine on Cognitive Decline, Cardiovascular Events, and Mortality in Dementia with Lewy Bodies: An up to 10-Year Follow-Up Study**

Hong Xu, MD PhD^1^, Annegret Habich, PhD^1,2^, Daniel Ferreira, PhD^1,3^, Londos Elisabet, MD PhD^1,4^, Eric Westman, PhD^1^, Maria Eriksdotter, MD PhD^1,5^

**TABLE OF CONTENTS**

**Supplementary Table e-1.** Definition of study covariates

**Supplementary Table e-2.** Definition of study outcomes

**Supplementary Table e-3** Baseline characteristics stratified by separate ChEIs within 3 months from an incident diagnosis of dementia with Lewy bodies.

**Supplementary Table e-4.** Mixed model output of estimated MMSE trajectories by treatment status.

**Supplementary Table e-5** Characteristics according to defined daily dosages (DDDs) of ChEIs in individuals with dementia with Lewy bodies.

**Supplementary Table e-6.** Number of events, incidence rates, and adjusted hazard ratios for the association between separate ChEIs and adverse clinical outcomes in the inverse probability of treatment weighted cohort.

**Supplementary Table e-7.** Mixed model output of estimated MMSE trajectories by treatment status censoring for ChEI or Memantine initiation during follow up (as-treated analysis).

**Supplementary Table e-8.** Number of events, incidence rates, and adjusted hazard ratios for the association between treatment status and adverse clinical outcomes in the inverse probability of treatment weighted cohort, censoring for ChEI or Memantine initiation during follow up (as-treated analysis).

**Supplementary Figure e-1.** **Balance of baseline characteristics before and after weighting.** Baseline characteristics before and after inverse probability of treatment weighting are shown. A standardized mean difference (SMD) of > 0.1 (dash line) indicates meaningful imbalance between groups.

Baseline characteristics before and after inverse probability of treatment weighting are shown. A standardized mean difference (SMD) of > 0.1 (dash line) indicates meaningful imbalance between groups.

**Supplementary Figure e-*2*.** **Balance of baseline characteristics before and after weighting.**

Baseline characteristics after inverse probability of treatment weighting are shown. A standardized mean difference (SMD) of > 0.1 (dash line) indicates meaningful imbalance between groups.

**Supplementary Figure e-*3*.** Mixed model output of estimated cognition trajectories by treatment status: A) slope of MMSE and B) MMSE score comparisons between different ChEIs at different timepoints. All ChEIs show an improvement at 1-5 years follow-up compared to non-users with the greatest improvement by donepezil and galantamine.

MMSE estimation is obtained in inverse probability of treatment weighted cohort, additionally adjusted with inverse probability of censoring weighting. The mixed model included treatments, visit time (year by using splines), treatment by visit time, number of MMSE measurements with an unstructured covariance matrix within treatment group for a repeated-measures covariance structure (random intercepts).

*P<0.05, **p<0.01, ***p<0.001

**Supplementary Figure e-4** Dose response of ChEI using cubic splines with MMSE changes. A) Starting ChEIs dose and MMSE trajectories. B) Distribution of dosages of ChEI within 3 months after incident dementia diagnosis.

The mixed model included ChEI treatment, visit time (year by using splines), treatment by visit time, and number of MMSE measurements further adjusted for inverse probability of treatment weighting and inverse probability of censoring weighting. Reference was set at lowest DDD value 0.05.

**Supplementary Figure e-5.** Cumulative incidence (A) and weighted hazard ratio curves (B) for all-cause mortality and major adverse cardiovascular events (MACE; composite of myocardial infarction, congestive heart failure or [stroke](https://www.sciencedirect.com/topics/medicine-and-dentistry/brain-ischemia)) for ChEIs and memantine versus non-use. Areas represent 95% confidence intervals (CIs).

**Supplementary Figure e-*6*** Subgroup analysis of A) sex and B) age with MMSE changes

**Supplementary Table e-1.** Definition of study covariates

| **Demographic characteristic** | **Definition** |  |
| --- | --- | --- |
| Age | Measured at index date |  |
| Sex | Male/female |  |
| MMSE score | Measured at dementia diagnosis date |  |
| Memory clinics visit | Where the dementia diagnosis was issued |  |
| Living situation | Living alone or with partner |  |
| Nursing home care | Living in nursing home |  |
| Dementia basic workups | Clock test, blood test, MMSE test and CT/MRI |  |
| **Comorbidities** | **ICD-10 codes** | **ATC codes** |
| Chronic kidney disease | I120, I131, N03[2-7], N05[2-7], N18, N19, N250, T856, T857, Z49[0-2], Z940, Z992 |  |
| Hypertension | I10-15 |  |
| Diabetes mellitus | E10-14 | A10A, A10B |
| Myocardial infarction | I21, I22, I252 |  |
| Congestive heart failure | I099, I110, I130, I132, I255, I420, I425-429, I43, I50, P290 |  |
| Atrial Fibrillation | I48 |  |
| Cardiac Pacemaker | Z950 |  |
| Atrial-Ventricular block | I440-443 |  |
| Bradycardia | R001 |  |
| Peripheral vascular disease | I70, I71, I731, I738, I739, I771, I790, I792, K551, K558, K559, Z958, Z959 |  |
| Cerebrovascular disease | G45-46, H340, I60-69 |  |
| Stroke | H341, I60, I61, I63, I64, I69 |  |
| Chronic pulmonary disease | I278, I279, J40-47, J60-67, J684, J701, J703 |  |
| Rheumatic disease | M05, M06, M315, M32, M33, M34, M351, M353, M360 |  |
| Peptic ulcer disease | K25-K28 |  |
| Malignant cancer | **C00-C97** |  |
| Liver disease | K70-K77 |  |
| Alcohol abuse | E244,F10,G312,G621,G721,I426,K292,K70,K860,O354,P043,Q860,T51,Y90,Y91,Z502,Z714 |  |
| Fracture | S12, S22, S32, S42, S52, S62, S72, S82, S92, M90 |  |
| Hearing loss | H90, H91, H833 |  |
| Depression | F32, F33, F204 |  |
| **Medication** |  | **ATC codes** |
| ACEI/ARBs |  | C09 |
| Beta blocking agents |  | C07 |
| Calcium channel blockers |  | C08 |
| Diuretics |  | C03 |
| Statins |  | C10 |
| NSAIDs |  | M01A |
| Antithrombotics |  | B01 |
| Anxiolytics |  | N05B |
| Hypnotics |  | N05C |
| Antipsychotics |  | N05A |
| Antidepressants |  | N06A |
| **Anti-dementia drugs** |  |  |
| Memantine |  | N06DX01 |
| Donepezil |  | N06DA02 |
| Rivastigmine |  | N06DA03 |
| Galantamine |  | N06DA04 |
| Calendar year | Measured at index date |  |

Abbreviations: MMSE, Mini-Mental State Examination; ACEI, angiotensin-converting enzyme inhibitors; ARB, angiotensin receptor blockers; NSAIDs, nonsteroidal anti-inflammatory drugs.

**Supplementary Table e-2.** Definition of study outcomes

| **Outcomes** | **Definition** |
| --- | --- |
| MMSE score | Measured at baseline and annual follow ups |
| Deaths | From death registry |
|  | **ICD-10 codes** |
| Major adverse cardiovascular events (MACE) | I099, I110, I130, I132, I255, I420, I425-429, I43, I50, P290  I21, I22, I252  H341, I60, I61, I63, I64, I69 |
| Congestive heart failure | I099, I110, I130, I132, I255, I420, I425-429, I43, I50, P290 |
| Myocardial infarction | I21, I22, I252 |
| Stroke | H341, I60, I61, I63, I64, I69 |

Abbreviations: MMSE, Mini-Mental State Examination.

#### **Supplementary Table e-3** Baseline characteristics stratified by separate ChEIs within 3 months from an incident diagnosis of dementia with Lewy bodies.

|  | Donepezil  (n=174) | Rivastigmine  (n=555) | Galantamine  (n=85) | p-value |
| --- | --- | --- | --- | --- |
| **Demographics** |  |  |  |  |
| Age, mean (SD) | 77.0 (6.8) | 76.0 (6.7) | 76.0 (6.4) | 0.26 |
| Female | 70 (40.2%) | 194 (35.0%) | 36 (42.4%) | 0.24 |
| MMSE baseline, mean (SD) | 21.9 (4.1) | 22.1 (4.3) | 22.7 (4.2) | 0.39 |
| MMSE strata |  |  |  | 0.53 |
| 10-19 | 45 (25.9%) | 139 (25.0%) | 21 (24.7%) |  |
| 20-24 | 83 (47.7%) | 241 (43.4%) | 33 (38.8%) |  |
| >25 | 46 (26.4%) | 175 (31.5%) | 31 (36.5%) |  |
| Specialist clinic | 160 (92.0%) | 519 (93.5%) | 83 (97.6%) | 0.21 |
| Living alone | 62 (35.6%) | 182 (32.8%) | 35 (41.2%) | 0.29 |
| Nursing home | 12 (6.9%) | 36 (6.5%) | 6 (7.1%) | 0.97 |
| **Comorbidities** |  |  |  |  |
| Chronic kidney disease | 7 (4.0%) | 20 (3.6%) | 0 (0.0%) | 0.19 |
| Hypertension | 67 (38.5%) | 221 (39.8%) | 35 (41.2%) | 0.91 |
| Diabetes | 17 (9.8%) | 70 (12.6%) | 13 (15.3%) | 0.41 |
| Myocardial infarction | 8 (4.6%) | 48 (8.6%) | 7 (8.2%) | 0.21 |
| Congestive heart failure | 18 (10.3%) | 36 (6.5%) | 5 (5.9%) | 0.20 |
| Atrial fibrillation | 27 (15.5%) | 86 (15.5%) | 6 (7.1%) | 0.11 |
| Cardiac pacemaker | 8 (4.6%) | 22 (4.0%) | 4 (4.7%) | 0.91 |
| Atrial-Ventricular block | 5 (2.9%) | 13 (2.3%) | 1 (1.2%) | 0.70 |
| Bradycardia | 3 (1.7%) | 6 (1.1%) | 0 (0.0%) | 0.46 |
| Peripheral vascular disease | 11 (6.3%) | 20 (3.6%) | 2 (2.4%) | 0.20 |
| Stroke | 10 (5.7%) | 47 (8.5%) | 4 (4.7%) | 0.29 |
| Chronical pulmonary disease | 13 (7.5%) | 47 (8.5%) | 5 (5.9%) | 0.69 |
| Rheumatic diseases | 5 (2.9%) | 28 (5.0%) | 1 (1.2%) | 0.16 |
| Peptic ulcers disease | 3 (1.7%) | 15 (2.7%) | 2 (2.4%) | 0.77 |
| Malignant cancer within 5y | 13 (7.5%) | 57 (10.3%) | 8 (9.4%) | 0.55 |
| Liver disease | 1 (0.6%) | 4 (0.7%) | 2 (2.4%) | 0.28 |
| Alcohol abuse | 1 (0.6%) | 11 (2.0%) | 2 (2.4%) | 0.41 |
| Fractures | 44 (25.3%) | 128 (23.1%) | 22 (25.9%) | 0.75 |
| Hearing loss | 21 (12.1%) | 50 (9.0%) | 12 (14.1%) | 0.23 |
| Depression | 18 (10.3%) | 59 (10.6%) | 9 (10.6%) | 0.99 |
| **Medication** |  |  |  |  |
| ACEI/ARB | 56 (32.2%) | 186 (33.5%) | 27 (31.8%) | 0.92 |
| β-blocker | 54 (31.0%) | 175 (31.5%) | 17 (20.0%) | 0.10 |
| Calcium channel blocker | 42 (24.1%) | 118 (21.3%) | 21 (24.7%) | 0.62 |
| Diuretics | 53 (30.5%) | 157 (28.3%) | 20 (23.5%) | 0.51 |
| Statins | 67 (38.5%) | 188 (33.9%) | 26 (30.6%) | 0.39 |
| NSAIDs | 19 (10.9%) | 62 (11.2%) | 12 (14.1%) | 0.71 |
| Antithrombotic | 104 (59.8%) | 376 (67.7%) | 59 (69.4%) | 0.12 |
| Antixiolytics | 44 (25.3%) | 117 (21.1%) | 14 (16.5%) | 0.25 |
| Hyponotics | 39 (22.4%) | 146 (26.3%) | 19 (22.4%) | 0.49 |
| Antipsychotics | 30 (17.2%) | 109 (19.6%) | 12 (14.1%) | 0.42 |
| Antidepressants | 64 (36.8%) | 229 (41.3%) | 35 (41.2%) | 0.57 |

Abbreviations: ChEI, acetylcholinesterase inhibitors; MMSE, Mini-Mental State Examination; ACEi, angiotensin-converting enzyme inhibitors; ARB, angiotensin receptor blockers. NSAIDs, nonsteroidal anti-inflammatory drugs.

#### **Supplementary Table e-4. Mixed model output of estimated MMSE trajectories by treatment status.**

| **2A) MMSE estimation,** β Coefficient **(95%CI)** | | | | |
| --- | --- | --- | --- | --- |
| Treatment status | None  (n=148) | Donepezil (n=174) | Rivastigmine  (n=555) | Galantamine  (n=85) |
| Baseline | 22.12(21.20 to 23.04) | 21.62(20.72,22.54) | 21.96(21.48,22.45) | 21.73(20.46 to 22.99) |
| Year 1 | 19.69(18.07 to 21.31) | 21.70(20.81,22.58) | 21.21(20.69,21.73) | 22.05(21.07 to 23.04) |
| Year 2 | 17.26(14.07 to 20.45) | 21.77(20.00,23.54) | 20.46(19.40,21.52) | 22.37(20.45 to 24.29) |
| Year 3 | 14.83(9.98 to 19.67) | 21.85(19.04,24.65) | 19.71(18.05,21.37) | 22.69(19.56 to 25.82) |
| Year 4 | 12.40 (5.88 to 18.92) | 21.92 (18.05,25.78) | 18.96 (16.67,21.24) | 23.01 (18.62 to 27.39) |
| Year 5 | 9.97(1.77 to 18.17) | 21.99(17.05,26.94) | 18.20(15.30,21.10) | 23.33(17.66 to 27.99) |
| MMSE slope | -2.43(-4.12 to -0.73) | 0.07(-1.01,1.16) | -0.75(-1.38, -0.12) | 0.32(-0.97 to 1.62) |

| **2B) Difference in MMSE,** β Coefficient **(95%CI)** | | | | | | |
| --- | --- | --- | --- | --- | --- | --- |
| Treatment  status | Donepezil  vs None | Rivastigmine  vs None | Galantamine  vs None | Rivastigmine  vs Donepezil | Galantamine  vs Donepezil | Galantamine  vs Rivastigmine |
| Baseline | -0.50(-1.77,0.77) | -0.16(-1.18,0.86) | -0.39(-1.93,1.15) | 0.34(-0.68,1.35) | 0.11(-1.44,1.65) | -0.23(-1.57,1.11) |
| Year 1 | 2.01(0.16,3.85)* | 1.52(-0.19,3.23) | 2.36(0.46,4.26)* | -0.49(-1.52,0.54) | 0.35(-0.97,1.67) | 0.84(-0.27,1.95) |
| Year 2 | 4.51(0.86,8.16)* | 3.20(-0.17,6.56) | 5.11(1.38,8.84)** | -1.31(-3.37,0.74) | 0.60(-2.01,3.21) | 1.91(-0.27,4.10) |
| Year 3 | 7.01(1.42,12.61)* | 4.88(-0.25,10.00) | 7.86(2.09,13.63)** | -2.14(-5.39,1.11) | 0.85(-3.34,5.03) | 2.98(-0.54,6.50) |
| Year 4 | 9.52(1.94,17.10)* | 6.55(-0.35,13.46) | 10.61(2.75,18.47)** | -2.96(-7.44,1.51) | 1.09(-4.73,6.92) | 4.06(-0.85,8.96) |
| Year 5 | 12.02(2.44,21.60)* | 8.23(-0.47,16.93) | 13.36(3.39,23.33)** | -3.79(-9.50,1.92) | 1.34(-6.14,8.82)* | 5.13(-1.19,11.44) |

# Estimation is obtained in inverse probability of treatment weighted cohort, additionally adjusted with inverse probability of censoring weighting (IPCW, we considered the potential effects of general attrition from those loss to follow-up due to drop-out or to the presence of a competing risk of death. The cohort was weighted for the following covariates: calendar year of diagnosis, age, sex , MMSE score at diagnosis, whether the diagnosis was issued at a memory clinic, whether the patient was living alone or in a nursing home, dementia basic workups (clock test, blood test, MMSE test and CT/MRI), comorbidities (alcohol abuse, acute kidney injury, atrial fibrillation, AV block, bradycardia, cancer, cerebrovascular disease, congestive heart failure, chronic kidney disease, chronic pulmonary disease, depression, diabetes, fracture, hearing loss, hypertension, liver disease, myocardial infarction, peptic ulcer disease, peripheral vascular disease, rheumatic disease, smoking, and stroke), presence of a cardiac pacemaker and ongoing medications (angiotensin-converting enzyme inhibitors /angiotensin receptor blockers (ACEI/ARBs), antidepressants, antipsychotics, antithrombotic, anxiolytics, β-blockers, calcium channel blocker, diuretics, hypnotics, nonsteroidal anti-inflammatory drugs, and statins).

The mixed model included treatments, follow-up time (year by using splines), treatment by follow-up year, number of performed MMSE assessments with an unstructured covariance matrix within treatment group for a repeated-measures covariance structure (random intercepts).

#### *P<0.05, **p<0.01, ***p<0.001

#### **Supplementary Table e-5** Characteristics according to defined daily dosages (DDDs) of ChEIs in individuals with dementia with Lewy bodies.

|  | **DDD 0.1-0.5** | **DDD 0.6-1** | **DDD 1.1-2** | p-value |
| --- | --- | --- | --- | --- |
| N | 184 | 268 | 362 |  |
| **Demographics** |  |  |  |  |
| Age, mean (SD) | 77.4 (6.1) | 76.0 (7.3) | 75.8 (6.5) | **0.02** |
| Female | 71 (38.6%) | 106 (39.6%) | 123 (34.0%) | 0.31 |
| MMSE baseline, mean (SD) | 21.6 (4.1) | 22.2 (4.4) | 22.3 (4.2) | 0.23 |
| MMSE strata |  |  |  | 0.23 |
| 10-19 | 56 (30.4%) | 66 (24.6%) | 83 (22.9%) |  |
| 20-24 | 82 (44.6%) | 115 (42.9%) | 160 (44.2%) |  |
| >25 | 46 (25.0%) | 87 (32.5%) | 119 (32.9%) |  |
| Specialist clinic | 168 (91.3%) | 253 (94.4%) | 341 (94.2%) | 0.35 |
| Living alone | 80 (43.5%) | 92 (34.3%) | 107 (29.6%) | **0.01** |
| Nursing home | 9 (4.9%) | 27 (10.1%) | 18 (5.0%) | **0.02** |
| **Comorbidities** |  |  |  |  |
| Chronic kidney disease | 6 (3.3%) | 9 (3.4%) | 12 (3.3%) | 1.00 |
| Hypertension | 76 (41.3%) | 107 (39.9%) | 140 (38.7%) | 0.83 |
| Diabetes | 28 (15.2%) | 32 (11.9%) | 40 (11.0%) | 0.37 |
| Myocardial infarction | 18 (9.8%) | 23 (8.6%) | 22 (6.1%) | 0.25 |
| Congestive heart failure | 16 (8.7%) | 22 (8.2%) | 21 (5.8%) | 0.36 |
| Atrial fibrillation | 27 (14.7%) | 38 (14.2%) | 54 (14.9%) | 0.97 |
| Cardiac pacemaker | 11 (6.0%) | 9 (3.4%) | 14 (3.9%) | 0.36 |
| Atrial-Ventricular block | 6 (3.3%) | 9 (3.4%) | 4 (1.1%) | 0.12 |
| Bradycardia | 3 (1.6%) | 1 (0.4%) | 5 (1.4%) | 0.36 |
| Peripheral vascular disease | 6 (3.3%) | 9 (3.4%) | 18 (5.0%) | 0.49 |
| Stroke | 16 (8.7%) | 17 (6.3%) | 28 (7.7%) | 0.63 |
| Chronical pulmonary disease | 18 (9.8%) | 20 (7.5%) | 27 (7.5%) | 0.59 |
| Rheumatic diseases | 9 (4.9%) | 12 (4.5%) | 13 (3.6%) | 0.74 |
| Peptic ulcers disease | 7 (3.8%) | 5 (1.9%) | 8 (2.2%) | 0.39 |
| Malignant cancer within 5y | 19 (10.3%) | 19 (7.1%) | 40 (11.0%) | 0.23 |
| Liver disease | 2 (1.1%) | 0 (0.0%) | 5 (1.4%) | 0.17 |
| Alcohol abuse | 4 (2.2%) | 4 (1.5%) | 6 (1.7%) | 0.85 |
| Fractures | 39 (21.2%) | 66 (24.6%) | 89 (24.6%) | 0.63 |
| Hearing loss | 14 (7.6%) | 32 (11.9%) | 37 (10.2%) | 0.33 |
| Depression | 24 (13.0%) | 29 (10.8%) | 33 (9.1%) | 0.36 |
| **Medication** |  |  |  |  |
| ACEI/ARB | 63 (34.2%) | 95 (35.4%) | 111 (30.7%) | 0.42 |
| β-blocker | 53 (28.8%) | 87 (32.5%) | 106 (29.3%) | 0.62 |
| Calcium channel blocker | 39 (21.2%) | 55 (20.5%) | 87 (24.0%) | 0.54 |
| Diuretics | 56 (30.4%) | 67 (25.0%) | 107 (29.6%) | 0.34 |
| Statins | 63 (34.2%) | 89 (33.2%) | 129 (35.6%) | 0.81 |
| NSAIDs | 26 (14.1%) | 27 (10.1%) | 40 (11.0%) | 0.39 |
| Antithrombotic | 132 (71.7%) | 165 (61.6%) | 242 (66.9%) | 0.08 |
| Antixiolytics | 44 (23.9%) | 57 (21.3%) | 74 (20.4%) | 0.64 |
| Hyponotics | 60 (32.6%) | 70 (26.1%) | 74 (20.4%) | **0.01** |
| Antipsychotics | 45 (24.5%) | 50 (18.7%) | 56 (15.5%) | **0.04** |
| Antidepressants | 90 (48.9%) | 106 (39.6%) | 132 (36.5%) | **0.02** |
| **ChEI type** |  |  |  | **<0.001** |
| Donepezil | 16 (8.7%) | 52 (19.4%) | 106 (29.3%) |  |
| Rivastigmine | 154 (83.7%) | 189 (70.5%) | 212 (58.6%) |  |
| Galantamine | 14 (7.6%) | 27 (10.1%) | 44 (12.2%) |  |

DDD= Defined Daily Dosage. 1DDD=Donepezil 7.5mg, Galatamine 16mg, Rivastigmine 9mg

Abbreviations: ChEI, acetylcholinesterase inhibitors; MMSE, Mini-Mental State Examination; ACEi, angiotensin-converting enzyme inhibitors; ARB, angiotensin receptor blockers. NSAIDs, nonsteroidal anti-inflammatory drugs

#### **Supplementary Table e-6.** Number of events, incidence rates, and adjusted hazard ratios for the association between separate ChEIs and adverse clinical outcomes in the inverse probability of treatment weighted cohort.

|  | Events | Person time, years | ^1^Overall Incidence rate per 1000 py (95%CI) | ^2^HR (95%CI)at Year 1 | ^2^HR(95%CI)at Year 2 | ^2^HR(95%CI)at Year 3 | ^2^HR(95%CI)at Year 10 |
| --- | --- | --- | --- | --- | --- | --- | --- |
| **Hospitalization due to MACE** | | | | | | | |
| Donepezil (n=174) | 16(9%) | 491.75 | 32.54(19.93 to 53.11) | 0.92 (0.39,2.16) | 0.82 (0.32,2.11) | 0.70 (0.21,2.31) | 0.48  (0.06, 3.88) |
| Rivastigmine(n=555) | 52(1%) | 1623.21 | 32.04(24.41 to 42.04) | 0.99 (0.47,2.07) | 0.85 (0.38,1.89) | 0.69 (0.25,1.85) | 0.40  (0.07,2.32) |
| Galantamine(n=85) | 13(15%) | 275.14 | 47.25(27.43 to 81.37) | 1.20 (0.51,2.87) | 1.29 (0.52,3.25) | 1.39 (0.47,4.12) | 1.66  (0.28,9.96) |
| **Deaths** |  |  |  |  |  |  |  |
| Donepezil (n=174) | 98(56%) | 514.50 | 190.48(156.26 to 232.18) | 0.61  (0.36,1.07) | 0.92  (0.65 ,1.30) | 1.17  (0.84,1.63) | 2.63*  (1.17,5.92) |
| Rivastigmine(n=555) | 310(56%) | 1701.40 | 182.20(163.01 to 203.66) | 0.65*  (0.43,0.97) | 0.83  (0.62 ,1.10) | 0.96  (0.72,1.28) | 1.58  (0.81,3.09) |
| Galantamine(n=85) | 56(66%) | 289.05 | 193.74(149.10 to 251.74) | 0.59  (0.33,1.04) | 0.84  (0.56 ,1.26) | 1.04  (0.69,1.56) | 2.14  (0.92,4.97) |

^1^Incidence rates are presented as number of events per 1000 patient-years in unweighted cohort.

^2^Hazard ratio is obtained in the inverse probability of treatment weighted cohort with flexible parametric survival model. The cohort was weighted for the following covariates: calendar year of diagnosis, age, sex , MMSE score at diagnosis, whether the diagnosis was issued at a memory clinic, whether the patient was living alone or in a nursing home, dementia basic workups (clock test, blood test, MMSE test and CT/MRI), comorbidities (alcohol abuse, acute kidney injury, atrial fibrillation, AV block, bradycardia, cancer, cerebrovascular disease, congestive heart failure, chronic kidney disease, chronic pulmonary disease, depression, diabetes, fracture, hearing loss, hypertension, liver disease, myocardial infarction, peptic ulcer disease, peripheral vascular disease, rheumatic disease, smoking, and stroke), presence of a cardiac pacemaker and ongoing medications (angiotensin-converting enzyme inhibitors /angiotensin receptor blockers (ACEI/ARBs), antidepressants, antipsychotics, antithrombotic, anxiolytics, β-blockers, calcium channel blocker, diuretics, hypnotics, nonsteroidal anti-inflammatory drugs, and statins).

#### **Supplementary Table e-7.** Mixed model output of estimated MMSE trajectories by treatment status censoring for ChEI or Memantine initiation during follow up (as-treated analysis).

|  | **MMSE estimation,** β Coefficient **(95%CI)** | | | **Difference,** β Coefficient **(95%CI)** | | |
| --- | --- | --- | --- | --- | --- | --- |
| Estimation of MMSE | None  (n=148) | Memantine  alone(n=133) | ChEI  alone(n=814) | Memantine  vs None | ChEI  vs None | ChEI  vs Memantine |
| Baseline | 22.09(21.23 to 22.95) | 22.35(21.51 to 23.19) | 21.72(21.31 to 22.12) | 0.25(-0.93,1.44) | -0.38(-1.31,0.56) | -0.63(-1.55,0.29) |
| Year 1 | 18.26(16.21 to 20.31) | 19.13(17.45 to 20.81) | 21.56(20.08 to 22.04) | 0.87(-1.78,3.52) | 3.30(1.19,5.42)** | 2.44(0.68,4.19)** |
| Year 2 | 14.42(10.40 to 18.45) | 15.91(12.56 to 19.25) | 21.41(20.41 to 22.42) | 1.48(-3.75,6.71) | 6.99(2.83,11.14)** | 5.50(2.01,9.00)** |
| Year 3 | 10.59(4.54 to 16.65) | 12.69(7.61 to 17.77) | 21.25(19.67 to 22.84) | 2.10(-5.81,10.00) | 10.67(4.40,16.93)*** | 8.57(3.25,13.89)** |
| Year 5 | - | - | 20.95(18.19 to 23.71) | - | - | - |
| MMSE slope | -3.83(-5.89 to -1.77) | -3.22(-4.98 to -1.45) | -0.15(-0.75 to 0.44) |  |  |  |

# Estimation is obtained in inverse probability of treatment weighted cohort, additionally adjusted with inverse probability of censoring weighting (IPCW, we considered the potential effects of general attrition from those loss to follow-up due to drop-out or to the presence of a competing risk of death. The cohort was weighted for the following covariates: calendar year of diagnosis, age, sex , MMSE score at diagnosis, whether the diagnosis was issued at a memory clinic, whether the patient was living alone or in a nursing home, dementia basic workups (clock test, blood test, MMSE test and CT/MRI), comorbidities (alcohol abuse, acute kidney injury, atrial fibrillation, AV block, bradycardia, cancer, cerebrovascular disease, congestive heart failure, chronic kidney disease, chronic pulmonary disease, depression, diabetes, fracture, hearing loss, hypertension, liver disease, myocardial infarction, peptic ulcer disease, peripheral vascular disease, rheumatic disease, smoking, and stroke), presence of a cardiac pacemaker and ongoing medications (angiotensin-converting enzyme inhibitors /angiotensin receptor blockers (ACEI/ARBs), antidepressants, antipsychotics, antithrombotic, anxiolytics, β-blockers, calcium channel blocker, diuretics, hypnotics, nonsteroidal anti-inflammatory drugs, and statins).

The mixed model included treatments, follow-up time (year by using splines), treatment by follow-up year, number of performed MMSE assessments with an unstructured covariance matrix within treatment group for a repeated-measures covariance structure (random intercepts).

#### *P<0.05, **p<0.01, ***p<0.001

#### **Supplementary Table e-8.** Number of events, incidence rates, and adjusted hazard ratios for the association between treatment status and adverse clinical outcomes in the inverse probability of treatment weighted cohort, censoring for ChEI or Memantine initiation during follow up (as-treated analysis).

|  | **Events** | **Person time, years** | **Overall Incidence rate per 1000 py (95%CI)**^1^ | **HR (95%CI) at Year 1**^2^ | **HR**  **(95%CI)**  **at Year 2**^2^ | **HR**  **(95%CI) at Year 3**^2^ | **HR**  **(95%CI) at Year 10**^2^ |
| --- | --- | --- | --- | --- | --- | --- | --- |
| **Hospitalization due to MACE** | | | | | | | |
| None  (n=148) | 16  (11%) | 281 | 57.0  (34.9 to 93.1) | Ref | Ref | Ref | Ref |
| Memantine (n=133) | 9  (7%) | 235 | 38.3  (19.9 to 73.6) | 1.05  (0.35,3.16) | 0.63  (0.21,1.86) | 0.47  (0.13,1.69) | 0.24  (0.03,1.78) |
| ChEI  (n=814) | 81  (10%) | 1598 | 50.7  (40.8 to 63.0) | 1.76  (0.74,4.20) | 1.21  (0.63,2.30) | 0.97  (0.47,1.99) | 0.57  (0.16,2.07) |
| **Deaths** |  |  |  |  |  |  |  |
| None  (n=148) | 77  (52%) | 281 | 274.3  (219.4 to 342.9) | Ref | Ref | Ref | Ref |
| Memantine (n=133) | 69  (52%) | 235 | 293.7  (231.9 to 371.8) | 1.15  (0.78,1.69) | 1.41  (0.90,2.20) | 1.55  (0.93,2.59) | 2.10  (0.94,4.70) |
| ChEI  (n=814) | 464  (57%) | 1598 | 290.4  (265.2 to 318.1) | 1.11  (0.83,1.48) | 1.31  (0.94 ,1.81) | 1.41  (0.98,2.02) | 1.79  (1.05,3.07) |

^1^Incidence rates are presented as number of events per 1000 patient-years in unweighted cohort.

^2^Hazard ratio is obtained in the inverse probability of treatment weighted cohort with flexible parametric survival model. The cohort was weighted for the following covariates: calendar year of diagnosis, age, sex , MMSE score at diagnosis, whether the diagnosis was issued at a memory clinic, whether the patient was living alone or in a nursing home, dementia basic workups (clock test, blood test, MMSE test and CT/MRI), comorbidities (alcohol abuse, acute kidney injury, atrial fibrillation, AV block, bradycardia, cancer, cerebrovascular disease, congestive heart failure, chronic kidney disease, chronic pulmonary disease, depression, diabetes, fracture, hearing loss, hypertension, liver disease, myocardial infarction, peptic ulcer disease, peripheral vascular disease, rheumatic disease, smoking, and stroke), presence of a cardiac pacemaker and ongoing medications (angiotensin-converting enzyme inhibitors /angiotensin receptor blockers (ACEI/ARBs), antidepressants, antipsychotics, antithrombotic, anxiolytics, β-blockers, calcium channel blocker, diuretics, hypnotics, nonsteroidal anti-inflammatory drugs, and statins).

#### **Supplementary Figure e-1.** **Balance of baseline characteristics before and after weighting.**

#### Baseline characteristics before and after inverse probability of treatment weighting are shown. A standardized mean difference (SMD) of > 0.1 (dash line) indicates meaningful imbalance between groups.

**
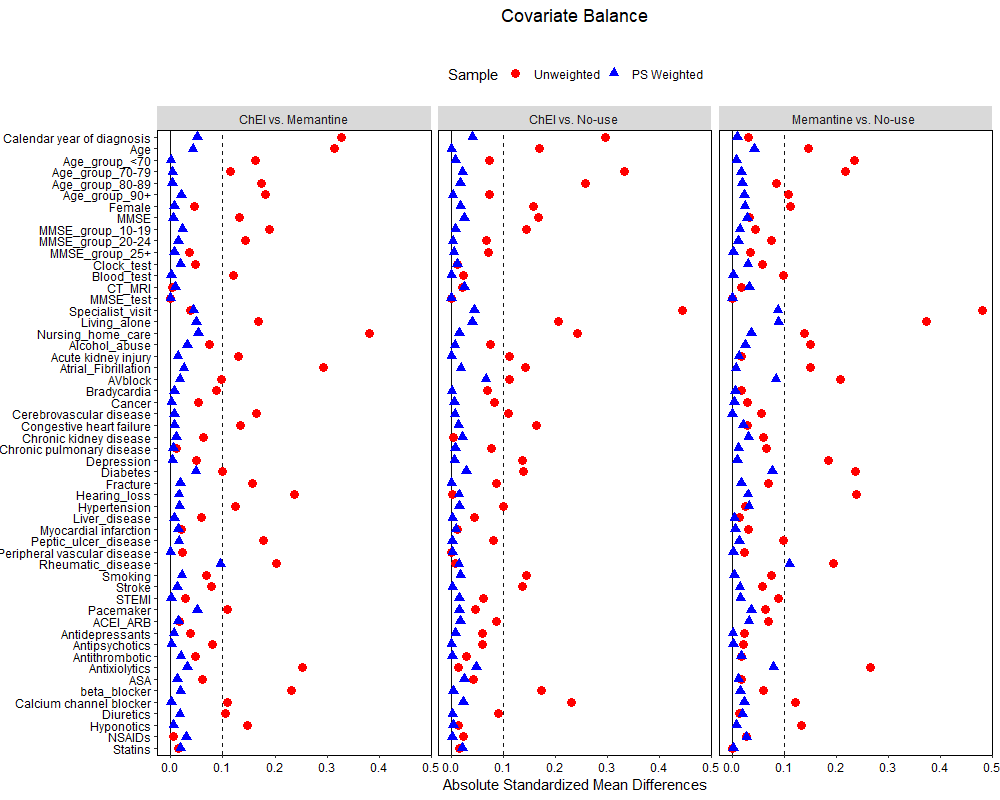
**

#### **Supplementary Figure e-2.** **Balance of baseline characteristics before and after weighting.**

#### Baseline characteristics after inverse probability of treatment weighting are shown. A standardized mean difference (SMD) of > 0.1 (dash line) indicates meaningful imbalance between groups.

#### **
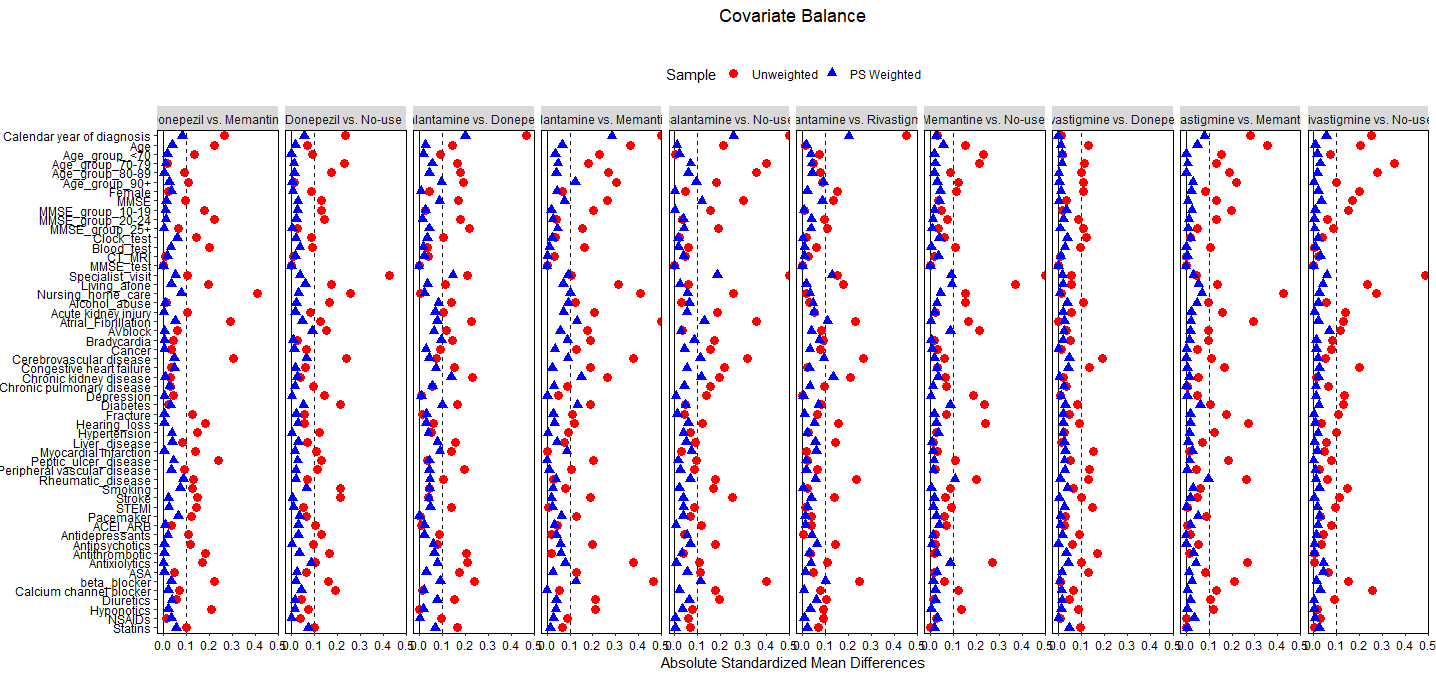
**

#### **Supplementary Figure e-3.** Mixed model output of estimated cognition trajectories by treatment status: A) slope of MMSE and B) MMSE score comparisons between different ChEIs at different timepoints. All ChEIs show an improvement at 1-5 years follow-up compared to non-users with the greatest improvement by donepezil and galantamine.

MMSE estimation is obtained in inverse probability of treatment weighted cohort, additionally adjusted with inverse probability of censoring weighting. The mixed model included treatments, visit time (year by using splines), treatment by visit time, number of MMSE measurements with an unstructured covariance matrix within treatment group for a repeated-measures covariance structure (random intercepts).

*P<0.05, **p<0.01, ***p<0.001

A)


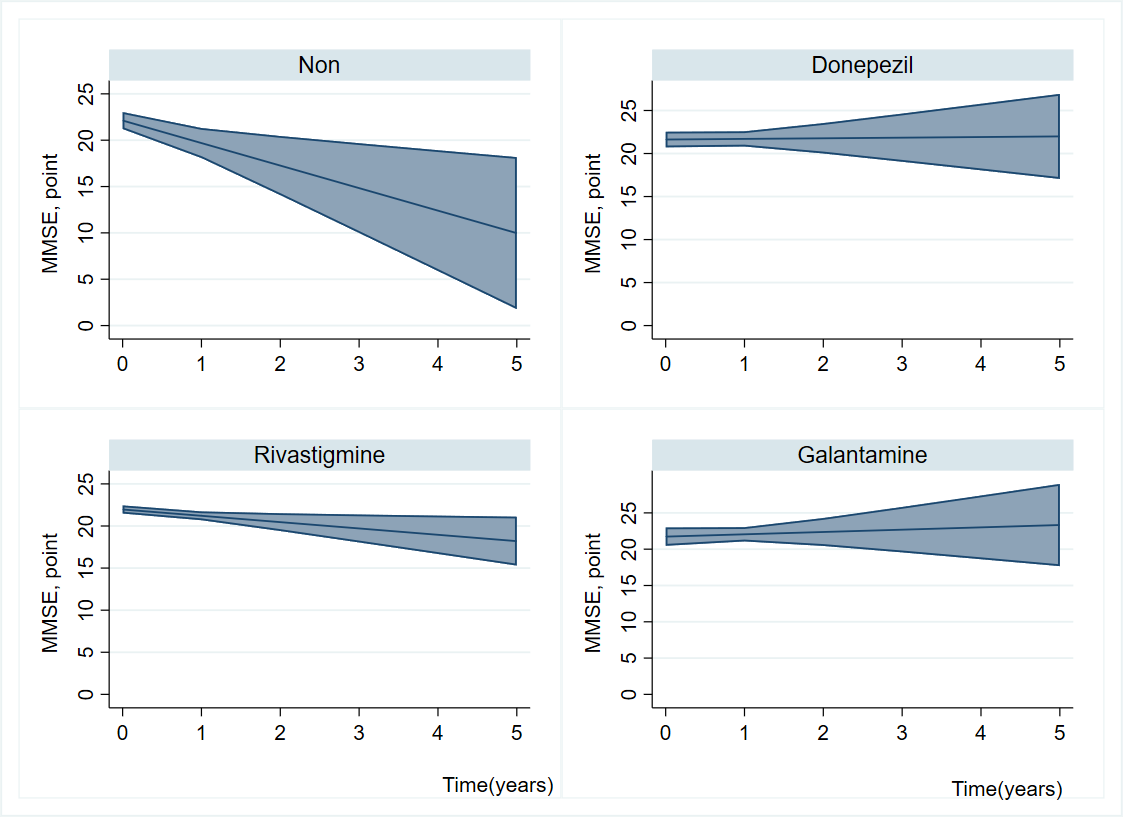


B)

####
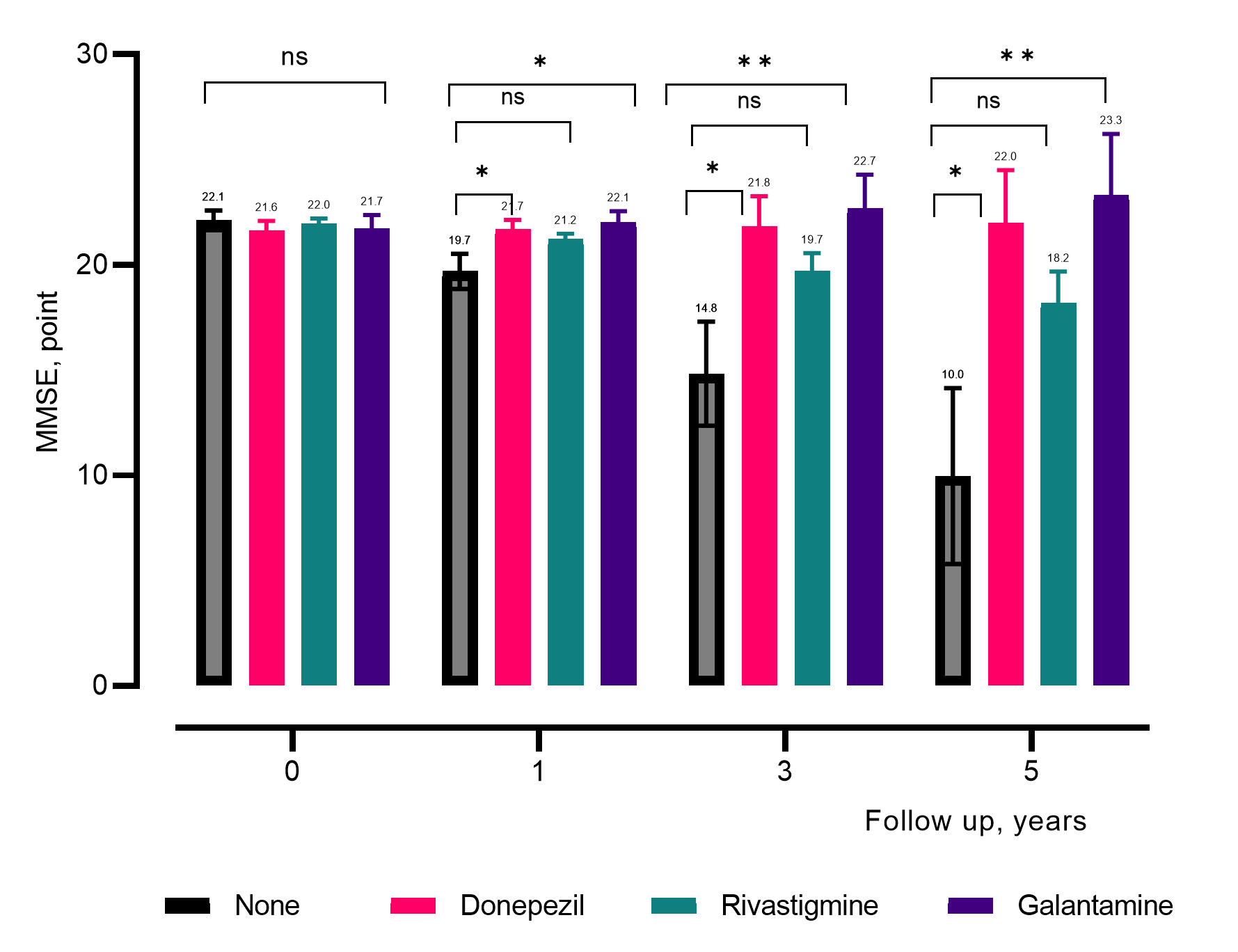


**Supplementary Figure e-4** Dose response of ChEI using cubic splines with MMSE changes. A) Starting ChEIs dose and MMSE trajectories. B) Distribution of dosages of ChEI within 3 months after incident dementia diagnosis.

The mixed model included ChEI treatment, visit time (year by using splines), treatment by visit time, and number of MMSE measurements further adjusted for inverse probability of treatment weighting and inverse probability of censoring weighting. Reference was set at lowest DDD value 0.05.

*
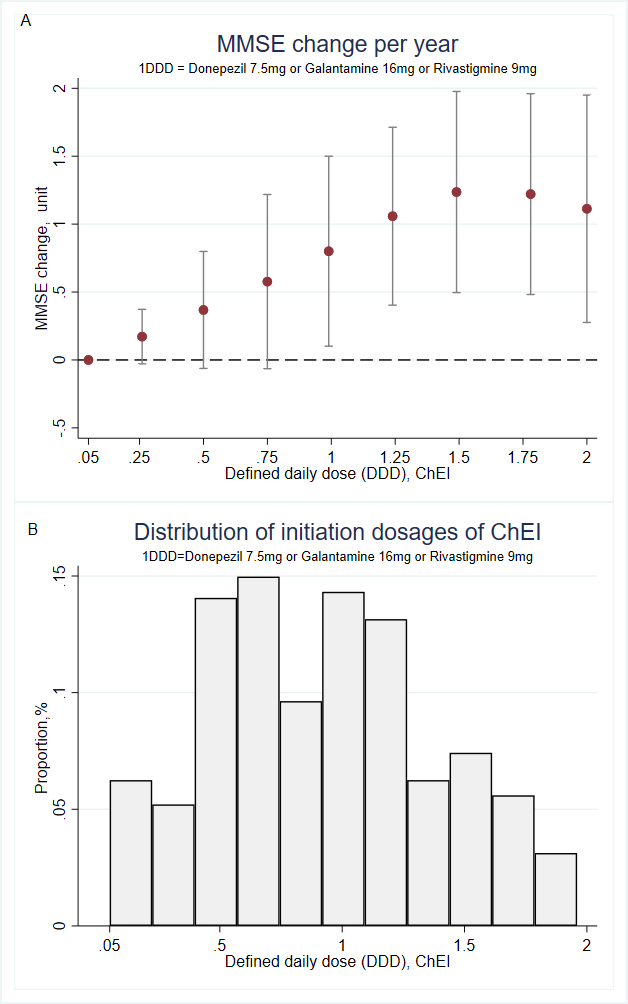
*

####

**Supplementary Figure e-5.** Cumulative incidence (A) and hazard ratio curves with 95% confidence intervals (CIs) (B) for all-cause mortality and major adverse cardiovascular events (MACE; composite of myocardial infarction, congestive heart failure or [stroke](https://www.sciencedirect.com/topics/medicine-and-dentistry/brain-ischemia)) for ChEIs and memantine versus non-use.

A)


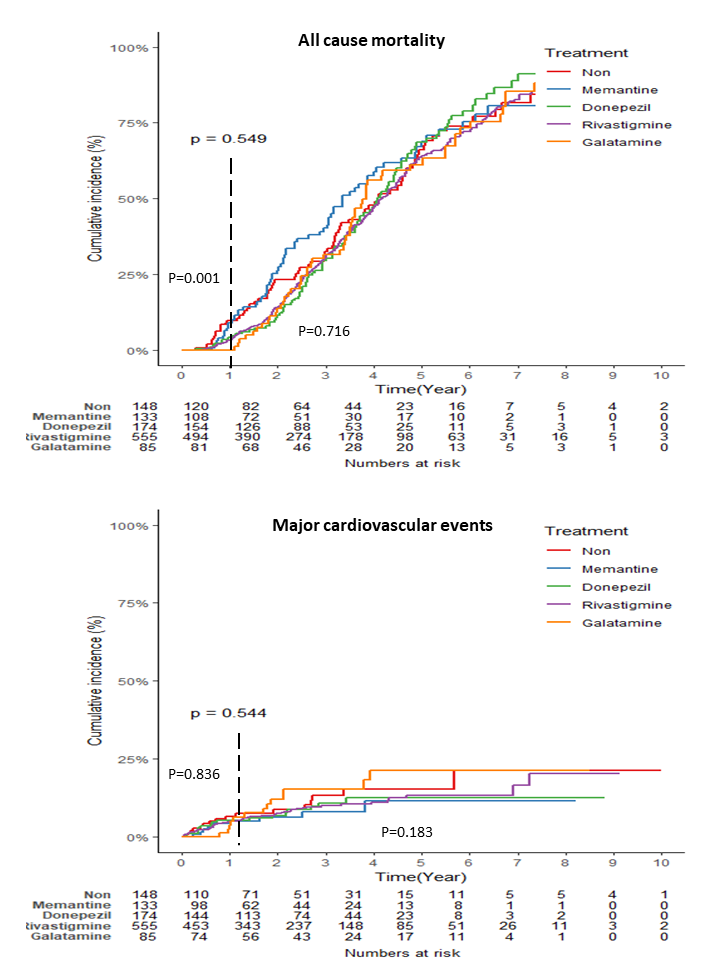


B)


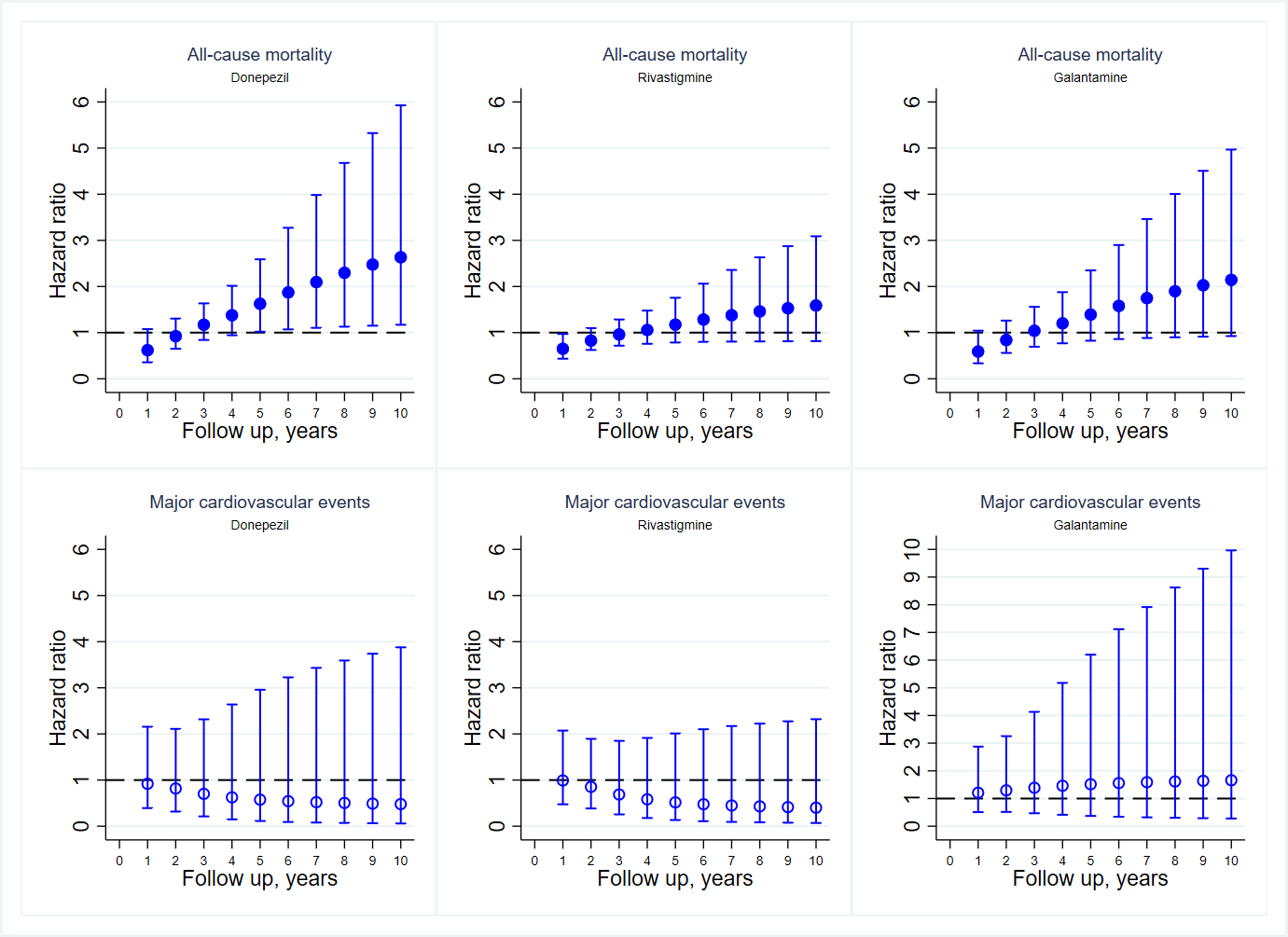


#### **Supplementary Figure e-6** Subgroup analysis of A) sex and B) age with MMSE changes

####
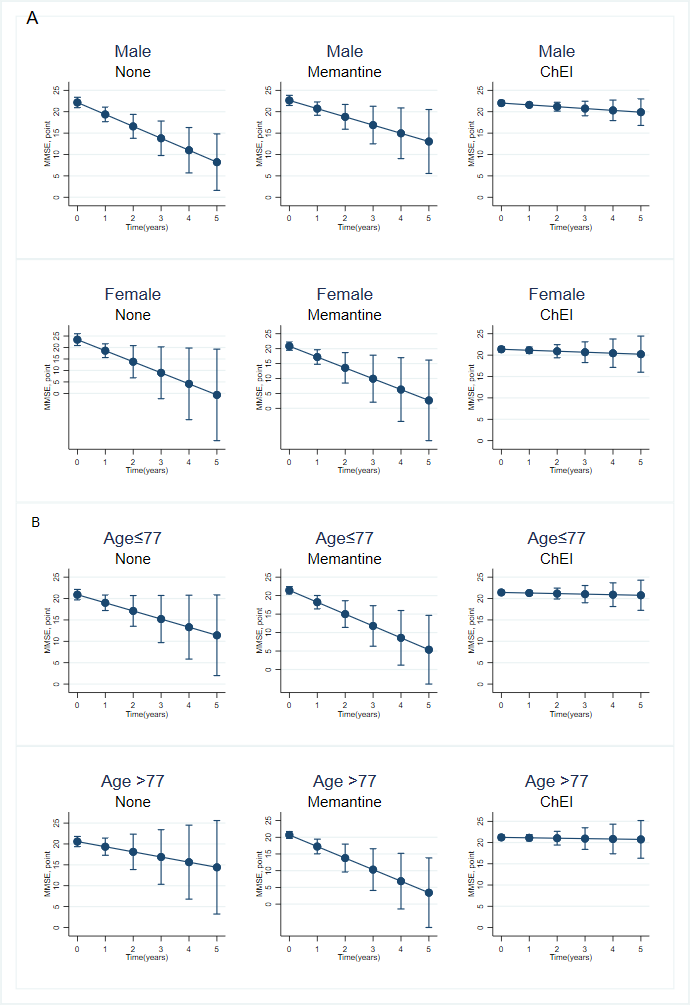

Supplement: Supplementary file 2 — Supporting Information [file ALZ-20-6740-s001.docx]
